# Traditional Mexican Medicinal Plants for Ocular Diseases: Ethnomedicinal Evidence, Pharmacological Relevance, and Research Gaps from Unexplored Sources

**DOI:** 10.3390/ph19101555

**Published:** 2026-10-01

**Authors:** Esteban Aceves-Fonseca, Santiago Xolalpa-Molina, Maira Huerta-Reyes

**Affiliations:** 1 Herbario Medicinal IMSS, Centro Médico Nacional Siglo XXI, Instituto Mexicano del Seguro Social, Avenida Cuauhtémoc # 330, Cuauhtémoc, Ciudad de México 06720, Mexico; eacevesfonseca@gmail.com (E.A.-F.); sxolalpa@yahoo.com (S.X.-M.); 2 Unidad de Investigación Médica en Enfermedades Nefrológicas, Hospital de Especialidades “Dr. Bernardo Sepúlveda Gutiérrez”, Centro Médico Nacional Siglo XXI, Instituto Mexicano del Seguro Social, Avenida Cuauhtémoc # 330, Cuauhtémoc, Ciudad de México 06720, Mexico

**Keywords:** ocular disorders, Mexican traditional medicine, IMSS Medicinal Herbarium, Asteraceae, *Matricaria chamomilla*, ophthalmological

## Abstract

**Background/Objectives**: Traditional medicinal knowledge remains underrepresented in pharmacological research because much of it is preserved in printed monographs, institutional collections, historical documents, and non-digitized ethnomedical repositories. This study aimed to document medicinal plants used in Mexican traditional medicine for ocular disorders and identify taxa of ethnomedicinal and pharmacological relevance. **Methods**: Three repositories were examined: the Medicinal Herbarium of the Mexican Institute of Social Security (IMSS), the open-access *Biblioteca de la Medicina Tradicional Mexicana*, and a pioneering undergraduate thesis addressing Mexican medicinal plants used for ophthalmological conditions. Data were manually extracted by two independent expert reviewers, with discrepancies resolved by consensus. Botanical nomenclature was updated using World Flora Online, and records were compared with previous Mexican ethnomedicinal information. Available pharmacological evidence was considered to contextualize the ethnomedical findings and identify taxa warranting further investigation. **Results**: The present contribution documented 232 ethnomedicinal records, corresponding to 174 species, 124 genera, and 57 botanical families. Asteraceae represented the predominant family, while *Matricaria chamomilla* was identified as the most frequently cited species. Notably, 165 ethnomedicinal records corresponding to 142 species identified in this work were absent from previous analysis of Mexican medicinal plants for ocular diseases, demonstrating the value of these repositories. Species such as *Argemone ochroleuca* exhibited notable ethnomedical relevance despite the limited pharmacological and clinical evidence currently available. **Conclusions:** The present work expands the existing ethnopharmacological knowledge of Mexican medicinal plants used for ocular diseases and highlights the value of integrating historical, institutional and digital sources into modern ethnopharmacological research. Furthermore, the findings identify and prioritize selected taxa for further pharmacological, toxicological, and formulations studies with potential ophthalmological applications.

## 1. Introduction

Mexico is considered one of the world’s megadiverse countries, harboring approximately 10% of the known flora [1]. Consequently, the study of its plant diversity, particularly its medicinal flora, is a national priority officially recognized by the Mexican government. Mexican legislation promotes the development, practice, strengthening and preservation of traditional medicine, including safeguarding the knowledge held by indigenous and rural communities [2].

Mexico is also characterized by remarkable biocultural diversity, encompassing 68 officially recognized indigenous linguistic groups whose traditional knowledge systems constitute an invaluable source of information regarding the medicinal uses of plants [3]. Over the past decades, substantial efforts have been devoted to documenting medicinal floras throughout the country, generating extensive ethnobotanical inventories that provide opportunities to investigate inter- and intracultural transmission of traditional knowledge, assess patterns of medicinal plants use, and identify culturally important species [4,5].

From a pharmaceutical perspective, ethnomedicinal knowledge represents an important starting point for the identification and prioritization of natural products with potential biological activity. Nevertheless, traditional use alone cannot establish pharmacological efficacy or therapeutic. Its principal value may lie in guiding the selection of plant species and preparations for subsequent phytochemical, pharmacological, toxicological, and clinical investigation [6]. This distinction is particularly relevant for medicinal plants used in ocular disorders because the anatomical and physiological characteristics of the eye impose specific requirements concerning efficacy, tissue compatibility, sterility, and safety [7].

Ocular diseases constitute a major public health challenge worldwide and are among the leading causes of visual impairment and reduced quality of life [8]. The increasing burden of ocular disorders, together with the need for accessible and safe therapeutic strategies, has stimulated interest in natural products as potential sources of pharmacologically active compounds [9]. Although conventional ophthalmic therapies are generally effective, some treatments may be associated with adverse effects [10], limited accessibility or poor patient adherence, highlighting the need to explore complementary therapeutic resources derived from medicinal plants.

The ocular surface is continuously exposed to environmental and physiological stressors, including ultraviolet radiation, airborne pollutants, allergens, humidity fluctuations and prolonged visual display terminal use, all of which may contribute to the development of inflammatory and degenerative ocular conditions [11]. Consequently, medicinal plants traditionally employed for ocular disorders represent a potentially valuable source of bioactive compounds for future pharmacological investigation.

Despite extensive ethnobotanical research conducted in Mexico, some ethnomedicinal records remain insufficiently accessible internationally. Many of these data are preserved in printed monographs, institutional archives or local publications [12].

This problem is particularly relevant to pharmacological research because the absence of digitized or searchable information can prevent potentially valuable traditional knowledge from being incorporated into contemporary evidence-based research [13]. Historical repositories may contain information on plant identity, geographical distribution, traditional indications, and routes of administration that is not readily retrievable through conventional electronic literature searchers.

To address this knowledge gap, we conducted an ethnopharmacological assessment based on a systematic analysis of Mexican ethnomedicinal sources documenting the traditional use of medicinal plants for ocular diseases. The sources selected for analysis were chosen because they represent repositories of traditional medical knowledge across the whole country and different cultural groups in Mexico. These sources included: (i) the Medicinal Herbarium of the Mexican Institute of Social Security (IMSS); (ii) the open-access *Biblioteca de la Medicina Tradicional Mexicana* (Library of Traditional Mexican Medicine); and (iii) a pioneering undergraduate thesis specifically devoted to ophthalmological ethnobotany in Mexico. Rather than merely compiling previously reported information, we analyzed and synthesized the ethnomedicinal records to characterize the diversity of medicinal plants used for ocular disorders, their taxonomic representation, and patterns of traditional use. These findings were subsequently contextualized within the available pharmacological evidence on the ocular-related activities of the principal documented species, with the aim of identifying and prioritizing plant species that may be the object of further pharmacological investigation for the development of potential approaches to ocular disorders.

Therefore, the objectives of this study were to (i) identify medicinal plant species reported in Mexican traditional medicine for ocular disorders; (ii) characterize their taxonomical and ethnomedicinal representation; (iii) identify records that were not included in previous published reports for Mexican medicinal plants for ocular diseases; and (iv) integrate the ethnomedicinal findings with available pharmacological information to identify species of particular interest for future natural product and ophthalmological drug research.

## 2. Materials and Methods

### 2.1. Medicinal Herbarium of the Mexican Institute of Social Security

The first ethnomedical source consulted in this work was the Medicinal Herbarium of the Mexican Institute of Social Security (IMSS), established in 1981 [14] by our research group. The IMSS Medicinal Herbarium is registered in the *Index Herbariorum* under the acronym (IMSSM) [15] and constitutes a specialized institutional collection focused on medicinal plants used in Mexican traditional medicine.

This specialized collection comprises more than 17,000 specimens of medicinal plants used in Mexican traditional medicine for the treatment of a wide range of conditions, including infectious diseases, endocrine-metabolic disorders, ocular and auditory disorders, tumours, male reproductive disorders, envenomations, poisonings, and children’s illnesses [16].

Despite the scientific, cultural, and social value of this collection, it remains undigitized, and much of its ethnomedical information has not been analyzed. Consequently, the present study used two previously published analyses of this collection, which are available in book format and, like the IMSS Medicinal Herbarium itself, have not been digitized. These books are: (1) a catalogue book containing information on the first 5000 specimens of the IMSS Medicinal Herbarium [14]; and (2) a book describing selected species from the IMSS Medicinal Herbarium IMSS and their distribution according to disease categories [16].

Because these sources are not fully searchable electronically, their examination required direct manual consultation of the printed material. This characteristic represents both a methodological limitation and a central rationale for the present study, as relevant ethnomedical information may remain inaccessible to conventional database-based approaches.

### 2.2. Biblioteca de la Medicina Tradicional Mexicana

In addition to the IMSS Medicinal Herbarium, another ethnomedical source consulted in this study was the book collection titled: *Atlas de las Plantas de la Medicina Tradicional Mexicana* (Atlas of Plants of Traditional Mexican Medicine). In this unique and specialized work on medicinal Mexican plants, more than 2000 monographs of medicinal species were compiled from local ethnobotanical data collected throughout the country. These monographs were originally published in 3 volumes in the year 1994 and included taxonomic, geographical, ecological, local use, pharmacological, anthropological, and historical data.

This work has recently been digitized and made publicly available as an open-access database, together with other ethnobotanical resources, including *La Medicina Tradicional de los Pueblos Indígenas de México* (Traditional Medicine of the Indigenous Peoples of Mexico), *Flora Medicinal Indígena de México* (Medicinal Flora of Mexico) and *Diccionario Enciclopédico de la Medicina Tradicional Mexicana* (Encyclopedic Dictionary of Mexican Traditional Medicine). Collectively, these works constitute the *Biblioteca de la Medicina Tradicional Mexicana* (Library of Traditional Mexican Medicine) [17].

Despite its digitization, this database does not provide a dedicated disease-indexing function that allows rapid and effective identification of all species associated with a particular disorder. Therefore, the only way to determine whether a particular plant species is used for a specific disease is by reviewing the monographs manually one by one, including the historical data content in the monographs, which also provides information about the medicinal use of the plants.

### 2.3. Historical Ophthalmological Ethnomedical Source

The third ethnomedical source consulted in this study was an undergraduate thesis in Biology which, to the best of our knowledge, had not been previously incorporated into published compilations of Mexican medicinal plants used for ophthalmological conditions.

This thesis work was conducted by Abigail Aguilar Contreras in 1974, founder of the IMSS Medicinal Herbarium (currently retired). This thesis is entitled: *Etnobotánica mexicana: plantas popularmente recomendadas para el tratamiento de afecciones oftalmológicas* (Mexican ethnobotany: plants popularly recommended for the treatment of ophthalmological conditions) [18], and, although it was not published, it has recently been made available online [19].

This historical source was considered particularly valuable because it was specifically devoted to ophthalmological ethnobotany and therefore provides information that may not be captured in broader medicinal-plant inventories.

### 2.4. Data Extraction and Botanical Validation

In all cases, the IMSS Medicinal Herbarium collection (examined from October 2025 to January 2026), the *Biblioteca de la Medicina Tradicional Mexicana* (examined from February to June 2026), and the pioneering undergraduate thesis (examined in July 2026), were examined manually by two independent expert reviewers without the use of automated virtual or electronic search tools. Any discrepancies in data extraction were resolved through discussion until consensus was reached.

Before data extraction, a predefined methodological framework was established, including the documentary sources to be consulted, search terms, eligibility criteria, procedures for handling duplicate records, data-extraction variables, and taxonomic validation procedures. Detailed information for each medicinal plant species was recorded to construct the results table. The three documentary sources were examined using a predefined set of Spanish terms both formal and related to ocular conditions, symptoms, and traditional terminology. Specific keywords included: *ojo*, *ojos*, *ocular*, *oftalmología*, *oftalmológico*, *conjunctivitis*, *cataratas*, *ceguera*, *legañas*, *chinguiñas*, *carnosidad*, *perrillas*, *vista*, *nubes*, *estrabismo*, *algodoncillo*, *retina*, *llanto*, *lloroso*, *córnea* and *leucoma*. Records identified through these terms were subsequently examined in their documentary context to determine they met the eligible criteria.

For the purposes of this study, an “ethnomedicinal record” was defined as a single documented report linking a medicinal plant species to an ocular disorder in one of the ethnomedical sources examined. Accordingly, the number of records does not necessarily correspond to the number of species, because the same species may be represented by more than one record when different reports, sources, ocular diseases, or traditional preparations were documented.

Records were eligible for inclusion when they met the following criteria:

1. Plant taxa documented as medicinally used for an ocular condition in at least one of the three selected repositories
2. Records referring to human traditional use, regardless of whether the source specified the preparation methods, plant part, or geographic origin
3. Records in which the reported plant could be identified at least to the genus level, with species-level identification retained whenever available.

Records were excluded when they met any of the following criteria:

1. Records lacking sufficient botanical information to establish the identity of the reported plant at least to the genus level
2. Non-plant materials, included animal-derived, mineral or other non-botanical remedies
3. Records referring to medicinal uses unrelated to ocular conditions or symptoms
4. Duplicate documentation of the same underlying ethnomedicinal report within the same documentary source.

Repeated mentions of the same plant species were not automatically considered duplicates. Because the selected repositories may document the same taxon in different ethnomedicinal contexts, records were retained as independent when they represent distinct documented reports, sources, ocular conditions, plant parts, or preparation methods.

**Figure 1:**
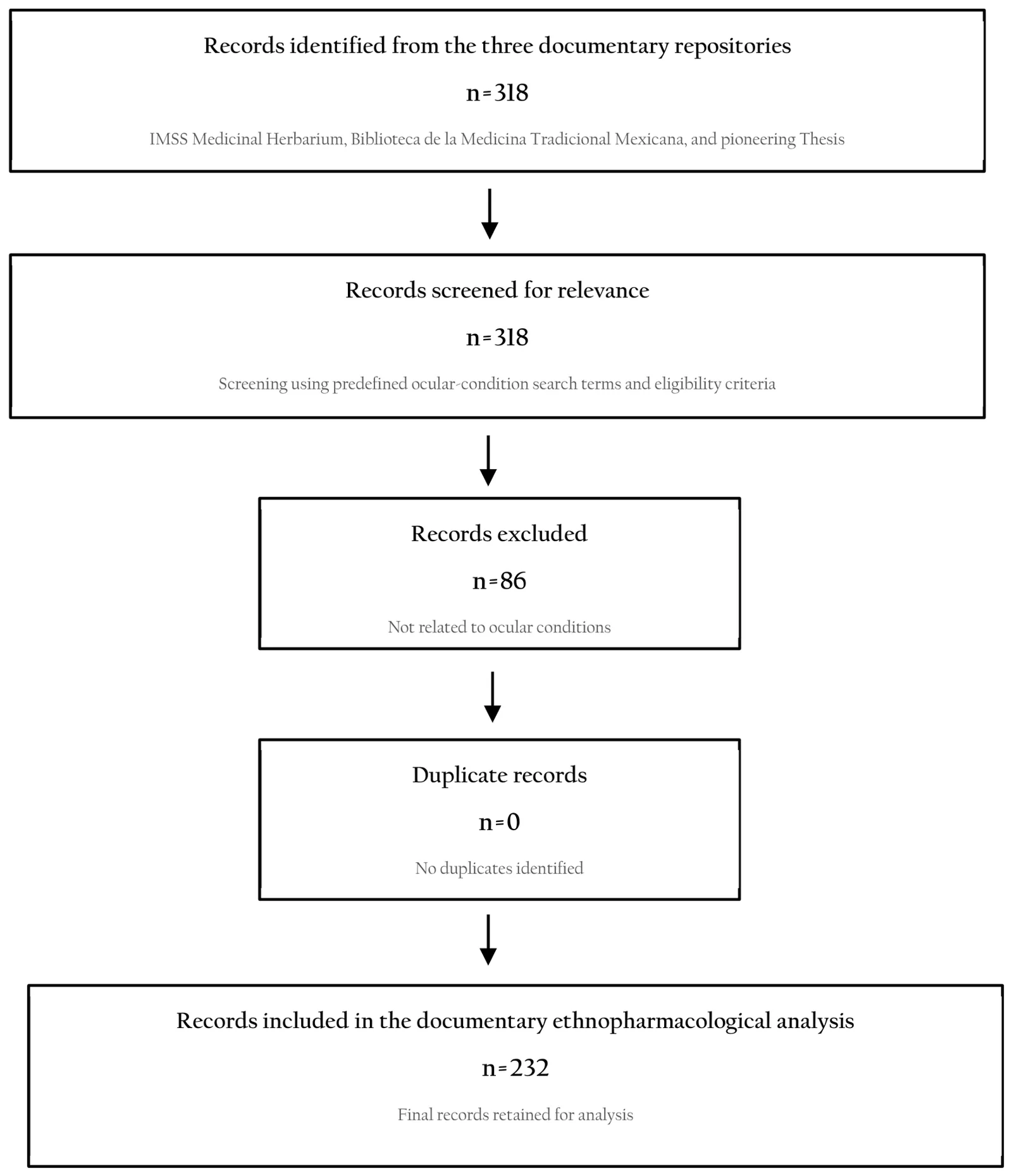
Flow diagram of the identification, screening, and selection of ethnomedicinal records for the documentary analysis of Mexican medicinal plants for ocular diseases.

The selection process, including the number of records identified, excluded, and retained for analysis, is summarized in Figure 1.

The validated botanical nomenclature of each species was verified according to The World Flora Online (WFO) [20]. The scientific names recorded in the original documentary sources were first transcribed as reported and subsequently checked against WFO database to standardize botanical nomenclature. For each record, the accepted name was identified, and relevant synonyms, outdated nomenclatural combinations, and spelling variants were reviewed. When a historical or synonymous name was reported in the original source, the currently accepted WFO name was used in the analytical dataset, while the original name was retained for traceability to the documentary source. Family and genus assignments were also standardized according to the corresponding WFO taxonomic treatment. Taxonomic changes were applied consistently across records from the three repositories. When the available documentary information was insufficient to establish the identity of a taxon with reasonable confidence, no taxonomic identification was inferred beyond the information supported by the original source.

### 2.5. Integration of Pharmacological Evidence

Published pharmacological information was considered as complementary evidence to contextualize the ethnomedicinal findings. The pharmacological literature was not subjected to a systematic or scoping review protocol; rather, relevant published evidence was examined to determine whether the medicinal taxa identified in the consulted repositories had previously been investigated experimentally or clinically. When available, the evidence was distinguished according to its experimental or clinical context, including *in vitro* studies, non-ocular animal or experimental models, ocular disease-related models, and clinical studies. Evidence derived from non-ocular models was considered general pharmacological evidence and was not interpreted as direct evidence of efficacy for ocular disorders. Similarly, evidence of antimicrobial, anti-inflammatory, antioxidant, or other biological activity obtained outside ocular models was not considered equivalent to evidence of therapeutic efficacy for ocular disorders. This approach was used to provide pharmacological context and identify research gaps, rather than to establish therapeutic efficacy or clinical readiness.

## 3. Results and Discussion

### 3.1. Overall Diversity of Medicinal Plants Used for Ocular Disorders

The present analysis documents 232 records of Mexican medicinal plants, belonging to 57 botanical families, 124 genera, and 174 plant species, used in the treatment of ocular diseases (Table 1) (after consideration of all criteria described in Figure 1), highlighting the remarkable diversity of traditional medicinal practices related to ocular health in Mexico.

**Figure 2:**
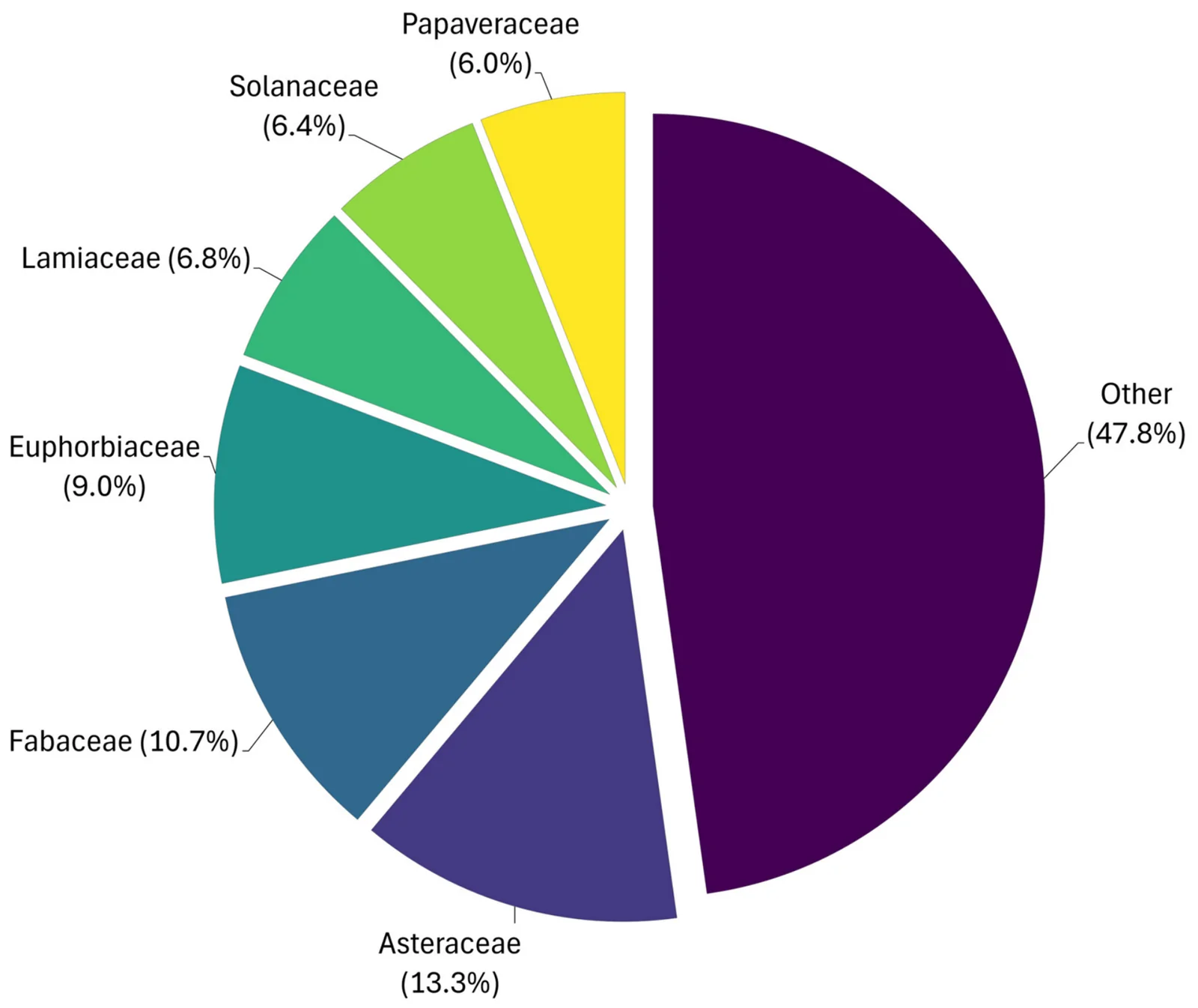
Botanical families most commonly used in Mexico for the treatment of eye diseases.

The predominance of the family Asteraceae in this work (31 records, 13.3%; 19 species) (Figure 2) is consistent with previous ethnobotanical studies conducted in Mexico and other regions of Latin America, where this family frequently ranks among the most represented [21,22,23,24].

The prominence of Asteraceae in Mexico can be partly attributable to the country’s role as an important center of diversification of this family and as the richest country for the family in the Neotropics. Its broad distribution, from sea level to the mountain regions, together with its high content of diverse secondary metabolites, including flavonoids, sesquiterpene lactones, polyacetylenes, alkaloids, monoterpenes, and essential oils, supports its ethnomedical relevance [25]. The wide spectrum of bioactivities reported for these compounds further reinforces the traditional importance of Asteraceae species in ophthalmic applications throughout the region, even though previous reports on Mexican traditional medicine have primarily recognized members of the Asteraceae family for their use in the treatment of gastrointestinal and respiratory disorders [26].

However, the predominance of a botanical family in terms of number of records should not be interpreted as evidence that its species possess greater pharmacological efficacy than species belonging to less represented families. Rather, family-level representation may reflect geographical distribution, availability, cultural preferences, historical documentation, and research effort. Consequently, pharmacological prioritization may be based on the convergence of ethnomedicinal relevance with experimental evidence and safety considerations [27].

### 3.2. Matricaria chamomilla L. (chamomile): Ethnomedicinal and Pharmacological Relevance

Within the family Asteraceae, *Matricaria chamomilla* L. (chamomile) emerged as the most frequently cited species in the present study (9 records), while eye problems and eye irritation were the most reported conditions (51 and 44 records, respectively). These findings demonstrate its prominent representation in the ethnomedicinal sources examined, although the frequency of traditional reports should not be interpreted as evidence of clinical efficacy.

The high frequency of citation identifies *M. chamomilla* as a priority species from an ethnopharmacological perspective, particularly because its traditional use is accompanied by a substantial body of experimental evidence describing anti-inflammatory, antimicrobial, and antioxidant activities. However, much of this experimental evidence derives from non-ocular models, and its relevance to specific ocular diseases remains to be established.

The main chemical constituents described in the flowers of chamomile comprise terpenoids, spiroethers, coumarins, and flavonoids including apigenin, quercetin, patuletin, luteolin, and their glucosides [28]. Among these compounds, apigenin has been identified as one of the most bioactive compounds due to its potent anti-inflammatory, antimicrobial, and antioxidant properties [29].

The strong anti-inflammatory activity of chamomile observed in animal models has been associated with the inhibition of the production of inflammatory mediators, including tumor necrosis factor alpha (TNF-α) and interleukin-1β (IL-1β) [30]. The essential oil profile of the flowers of chamomile includes terpenoids such α-bisabolol and its oxides, and azulenes such as chamazulene [31]. Chamomile essential oils are well known because of their antimicrobial properties against a wide spectrum of Gram-negative and Gram-positive bacteria, highlighting bacteriostatic effects against *Pseudomonas aeruginosa*, *Escherichia coli*, and *Staphylococcus aureus* [29]. In addition, their antifungal activity against *Candida albicans* and *Aspergillus* sp. species has been attributed to the presence of α-bisabolol, which shows strong inhibition on fungal growth [32,33]. Therefore, these pharmacological findings provide a rationale for further investigation of chamomile constituents for scientific support for its traditional medicinal use.

In Mexican traditional medicine, chamomile is primarily administered as ocular washes prepared from dried flower infusions as shown by our findings (Table 1), which is consistent with previous reports in the literature [34]. This route of traditional administration is particularly important from a pharmaceutical perspective. Although an aqueous infusion may contain biological active constituents, preparations intend for direct ocular application require stringent control of microbial contamination, chemical composition, concentration, pH and osmolarity. Therefore, the direct application of homemade preparations may pose microbiological risks if sterility is not ensured [35].

Available evidence regarding chamomile must be interpreted in light of the route and method of administration. Allergic and hypersensitivity reactions, including contact dermatitis and, less frequently, severe systemic reactions, have been reported following exposure to chamomile preparations, particularly in individuals sensitized to plants of the Asteraceae family [36,37]. Ophthalmic use requires special precautions, as the application of non-sterile chamomile infusions or preparations may entail additional limitations due to the lack of sterility and the risk of microbial contamination; consequently, they should not be considered equivalent to a sterile ophthalmic preparation [38].

Interestingly, despite the extensive ethnomedical background supporting the use of *M. chamomilla* in ocular conditions, clinical trials in humans remain scarce. Consequently, rigorous clinical research is needed to evaluate its efficacy, safety, optimal dosage, and potential pharmacological interactions.

### 3.3. Argemone ochroleuca: High Ethnomedicinal Relevance with Important Safety Gaps

Another interesting species in the present contribution is *Argemone ochroleuca*, which belongs to Papaveraceae family. This species was the second most frequently cited species in the present study, only with 2 reports less (7 reports) than *M. chamomilla* (Table 1).

In contrast with *M. chamomilla*, the species *A. ochroleuca* represents a relevant example of ethnomedical knowledge that, although well documented in several regions of Mexico [39], remains insufficiently explored from pharmacological and clinical perspectives. In the present analysis, its citation of ophthalmologic applications suggests a persistent traditional use, likely associated with its availability and perceived therapeutic efficacy in local contexts [40].

Phytochemical studies have demonstrated that species of the genus *Argemone* contain isoquinoline alkaloids, including sanguinarine, dihydrosanguinarine, and berberine, compounds widely recognized for their antimicrobial and anti-inflammatory properties [41,42]. These biological activities provide a basis for further investigation, in which scientific experimentation in relevant ocular models is therefore necessary to determine whether these activities translate in to measurable effects in ocular tissues.

In particular, sanguinarine, has shown antimicrobial activity against Gram-positive bacteria, which may be relevant in superficial ocular infections [43]. However, some of these alkaloids present in *A. ochroleuca* have been associated with toxic effects, mainly cytotoxic effects [44]. Nevertheless, the clinical evidence supporting the efficacy and safety of *A. ochroleuca* in the treatment of eye diseases is still pending. Most available data derived from ethnobotanical surveys or in vitro studies, limiting the ability to establish evidence-based therapeutic recommendations. This gap highlights the need for pharmacologic and clinical research in ocular models to evaluate the therapeutic potential and safety of this plant.

The metabolite composition, including alkaloids, in *A. ochroleuca*, may vary according to the plant material and extraction procedure. Recent analysis demonstrated differences in metabolite profiles between shoots and roots and among water, methanol, acetone, and dichloromethane extracts, indicating that both plant part and extraction solvents can influence the compound recovered [45]. Thus, comparisons of the chemical composition and potential biological effects of *A. ochroleuca* preparations should consider the plant material and extraction method used. Evidence concerning additional sources of variation, such as geographic origin and development stage, remains limited for *A. ochroleuca*.

### 3.4. Fabaceae and Euphorbiaceae: Underexplored Taxa and Safety Considerations

Other botanical families were also significantly represented in the present work such as Fabaceae (25 records, 10.7%; 19 species) and Euphorbiaceae (21 records, 9.0%; 15 species) (Figure 2).

The representation of Fabaceae is particularly interesting because the available literature on its traditional use of ocular disorders is comparatively limited. To the best of our knowledge, only some species of the genera *Abrus*, *Senna* and *Acacia* have shown biological activities potentially related to ophthalmological applications, as they have been reported to contain compounds with significant activity against multidrug-resistant strains of *Staphylococcus*, which are frequently implicated in ophthalmic infections such as conjunctivitis. Phytochemical investigations of these taxa have revealed the presence of phenolic compounds and flavonoids, including epicatechin and lupeol, which may contribute to antimicrobial effects [46,47,48]. However, none of these genera are represented in the present work. Thus, the 19 species of Fabaceae documented in the present study, which were not reported in the previous review in the context of ophthalmological diseases, represent a valuable source of priority taxa for further investigation, particularly through chemical characterization, ocular pharmacological studies and safety assessment (Table 1).

The genus *Euphorbia* (Euphorbiaceae) is particularly noteworthy in the present study, comprising 7 distinct species (Table 1). *Euphorbia* is an important genus due to its cosmopolitan distribution and because it is the third largest genus of flowering plants in the world [49]. The latex of *Euphorbia* species contents numerous biologically highly active natural products, which have been used in a wide range of medicinal applications, particularly diterpenes such as ingenanes, among which ingenol mebutate stands out, a compound used in the topical treatment of actinic keratosis [50].

Although the most recognized medicinal use of the genus *Euphorbia* refers to gastrointestinal affections, some species have also been used for the treatment of eye disorders, as well as for the management of blindness, cataracts, ear complaints and deafness, particularly on the American continent [51]. However, some species of *Euphorbia* have shown poisonous or toxic effects [52] especially in dermatological cases. Therefore, rigorous pharmacological and toxicological studies of the latex of species belonging to *Euphorbia* are still needed.

The pharmaceutical relevance of *Euphorbia* therefore illustrates both the opportunities and challenges associated with medicinal plants. The presence of pharmacologically active diterpenoids demonstrates the potential of the genus as a source of bioactive molecules, whereas the irritant and toxic properties reported for several species emphasize the need for rigorous safety evaluation before consideration of ocular applications.

#### Croton and the Importance of Ocular Safety

Also belonging to Euphorbiaceae, the genus *Croton* stands out because of its 7 records that represent also 7 different species in the present study. Phytochemical investigations have demonstrated that *Croton* species are a rich source of structurally diverse bioactive compounds, including terpenoids, alkaloids, flavonoids, and essential oils that exhibit antibacterial, antifungal, antioxidant, and anti-inflammatory activities in experimental models [53,54,55,56,57].

Nevertheless, the pharmacological evidence supporting ocular applications remains limited because many reported activities have been demonstrated in non-ocular in vitro assays. Moreover, the latex of several *Croton* species contains irritant and potentially cytotoxic metabolites [58,59]. These characteristics make ocular toxicology a particularly important component of any future investigations of this genus.

### 3.5. Other Important Botanical Families in Mexico in the Treatment of Ocular Diseases

Other well-represented families in the present work include Lamiaceae (16 records, 6.8%; 14 species), Solanaceae (15 records, 6.4%; 12 species), and Papaveraceae (14 records, 6.0%; 5 species) (Figure 2), all of which are widely recognized for their medicinal relevance.

In the case of Crassulaceae (7 records, 3.0%; 3 species), this family has been previously reported as the most represented in other studies of Mexican flora for the treatment of eye diseases [60]. Our present findings extend this previous evidence by documenting additional species of the genus *Sedum*, including *S. praealtum* and *S. dendroideum*, as well as the species *Kalanchoe pinnata*, that was not reported in the previous review for this family [60]. These findings illustrate the value of consulting historical and institutional repositories in parallel with previously published ethnobotanical studies.

Our results are consistent with those reported by Salazar-Gómez et al. (2023) [60], particularly regarding the prominence of *M. chamomilla* and the representation of several major botanical families. However, the two studies address complementary aspects of Mexican ethnomedicinal knowledge for ocular diseases and differ substantially in their source selection and methodological approach. The present study specifically targeted ethnomedical repositories that have remained insufficiently explored, including the undigitized IMSS Medicinal Herbarium, the *Biblioteca de la Medicina Tradicional Mexicana*, and a historical undergraduate thesis devoted specifically to ophthalmological ethnobotany. Consequently, the present study was not intended to reproduce the previous inventory. Indeed, 165 records identified in the present study corresponding to 142 species were not reported by Salazar-Gómez et al. (2023) [60], demonstrating that a substantial body of relevant information remain unexplored. Moreover, of these 165 additional records, 122 records were obtained from the *Biblioteca de la Medicina Tradicional Mexicana*, while 33 were obtained from the IMSS Medicinal Herbarium collection, and 10 from the pioneering undergraduate thesis. This distribution underscores the contribution of sources that are either insufficiently indexed or not readily accessible through conventional scientific databases. Nonetheless, rather than emphasizing the number of records that differ between studies, the present findings demonstrate that consultation of complementary historical and institutional repositories can recover ethnomedicinal information that is not necessarily captured in previously published inventories. This complementary approach is particularly relevant for documenting traditional uses that may remain inaccessible through conventional scientific databases and for identifying taxa that warrant further ethnopharmacological investigation.

### 3.6. Plant Parts and Geographic Distribution

**Figure 3:**
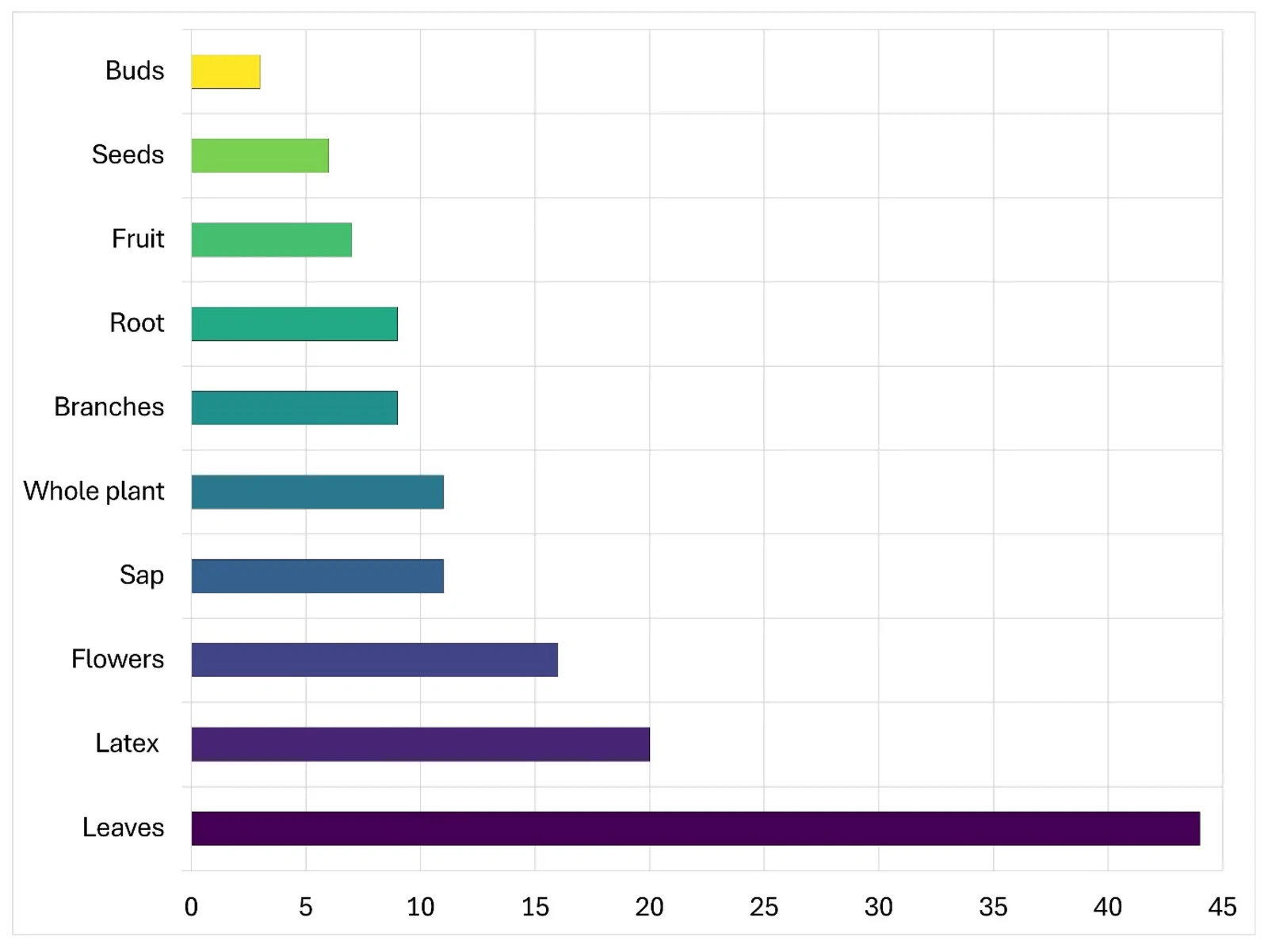
Parts of the plant most frequently reported for the treatment of ocular diseases in Mexico.

The predominance of leaves as the most used part of the plant (44 records) (Figure 3) may be explained by their accessibility, ease of collection, and high concentration of bioactive compounds.

Leaves are often prepared as infusions or decoctions, facilitating topical application in the form of eye washes or compresses [61].

Concerning geographic distribution, it is interesting to note that Puebla was the most represented state in this study with 83 records, followed by Oaxaca (57 records), Michoacán (49 records), and San Luis Potosí (48 records). This pattern does not necessarily reflect greater ethnomedical knowledge or higher plant diversity in these states; rather, it may be associated with historical and logistic factors that have influenced the development of ethnobotanical research in Mexico. In particular, the geographical proximity of these states to Mexico City, where many of the country’s most important ethnobotanical expeditions have originated, may have facilitated a greater research effort in nearby regions due to economic and operational constraints [4].

This trend is evident in states recognized for their biological diversity, such as Chiapas, which represented by only 18 records in the present study. The social and political factors, including periods of social unrest and armed conflict in certain regions of Chiapas, may have further constrained the development of ethnobotanical studies [62]. Similarly, Baja California Sur, one of the states located farthest from the country’s central region, showed a low representation (2 records).

Consequently, the observed distribution of records among states likely reflects, at least in part, differences in research effort rather than actual variations in traditional knowledge richness or plant diversity. These factors have contributed to an uneven distribution of research effort across the country, highlighting the need for further ethnobotanical research to achieve a more comprehensive understanding of Mexican traditional medicine across its diverse cultural and ecological regions.

### 3.7. Ethnomedical Repositories as Resources for Natural-Product Discovery

Collectively, these findings highlight the relevance of both institutional and digital repositories as sources of ethnopharmacological information.

The principal pharmaceutical relevance of these repositories lies in their capacity to generate hypotheses for natural product-discovery. Historical records may identify plants, plant parts, preparations, and therapeutic indications that can subsequently be investigated using modern phytochemical, pharmacological, toxicological, and pharmaceutical approaches.

In particular, the IMSS Medicinal Herbarium emerges as a significant and continuously expanding repository of ethnomedicinal knowledge. Its undigitized status, however, represents a major accessibility barrier. Digitization, taxonomic updating, standardized metadata, and disease-oriented indexing would facilitate integration of this information with contemporary pharmacological databases and could substantially increase the visibility of Mexican ethnomedicinal knowledge in international research.

Likewise, the *Biblioteca de la Medicina Tradicional Mexicana*, despite being freely accessible online, remains unexplored in terms of systematic analysis and data extraction, thus representing a valuable and untapped source of ethnobotanical information with considerable potential for future research.

The magnitude of the ethnomedicinal knowledge included in the present work is particularly relevant when considered in relation to the accessibility of the underlying sources. A substantial proportion of the information was retrieved from repositories that are not readily searchable through conventional scientific databases, demonstrating that historical and institutional ethnomedical collections may contain information of potential relevance to contemporary natural-product research.

## 4. Limitations

Several limitations should be considered when interpreting the findings of this study. First, the analysis was restricted to the three specific ethnomedicinal sources: the IMSS Medicinal Herbarium, the *Biblioteca de la Medicina Tradicional Mexicana*, and the historical thesis by Aguilar Contreras. Although these repositories provide complementary evidence, they do not encompass the full diversity of Mexican ethnomedicinal knowledge. Regional floras and other local or institutional sources may contain additional information that was not accessible for analysis in the present study. The accessibility and digitization sources also varied. While the *Biblioteca de la Medicina Tradicional Mexicana* is available in digital form, the IMSS Medicinal Herbarium collection has not yet been digitized. This may limit the completeness and reproducibility of future searches and highlights the importance of continued digitization standardized metadata and disease-indexed databases for Mexican ethnomedical collections.

Another limitation concerns the variability in terminology used to describe ocular disorders and symptoms. Traditional and historical records frequently employ vernacular expressions, regional terminology or nonspecific descriptions, which do not necessarily correspond to contemporary clinical diagnoses. Although an expanded set of Spanish search terms was used to maximize retrieval, terminology cannot always be mapped unambiguously onto modern ophthalmological categories. The reported uses should therefore be interpreted as documented ethnomedicinal indications rather than as confirmed clinical diagnosis.

A further limitation concerns the heterogeneity of the available pharmacological evidence. The identified taxa vary considerably in the amount, quality, and experimental relevance of published pharmacological information. Much of the available experimental evidence is derived from non-ocular models and therefore cannot be assumed to demonstrate efficacy in specific ocular diseases. Therefore, the pharmacological information presented in this study should be regarded as complementary context for interpreting the ethnomedicinal findings and for identifying research gaps rather than evidence of therapeutic efficacy.

The ethnomedicinal records analyzed in this study should be interpreted within the cultural and geographical diversity of Mexican traditional medicine. The selected repositories compile information generated in different regions and cultural contexts, including knowledge associated with indigenous and rural communities. However, the level of cultural information available was not consistent across the three sources. In particular, some historical records provide information without explicitly identifying the indigenous group, community, or language associated with the reported practice. The present study consequently does not aim to quantify or compare ethnomedicinal knowledge among specific cultural groups, but rather to recover and characterize documented traditional uses of medicinal plants for ocular diseases. This distinction is important because traditional medicinal knowledge is culturally and geographically situated and may vary among communities according to local ecological conditions, cultural practices, and terminology. Differences in the availability and historical documentation of ethnomedicinal information may also influence the apparent representation of regions or cultural context in the present dataset.

A further and particularly important limitation concerns the limited availability of evidence regarding the ocular safety of the medicinal plants identified in the consulted repositories. Although traditional use provides valuable information on cultural relevance and may help to prioritize taxa for further investigation, it should not be interpreted as evidence of ocular safety, efficacy, or therapeutic suitability. Importantly, this study does not make recommendations for the use, self-administration, or therapeutic application of any of the documented plants for ocular conditions. Rather, the reported uses are presented as ethnomedicinal evidence that may inform priorities for future pharmacological and toxicological research. This distinction is particularly relevant because some traditional practices involve preparations intended for direct ocular applications, for which potential risks include corneal toxicity, epithelial damage, inflammatory or hypersensitivity reactions, alterations in intraocular pressure, and microbial contamination. Moreover, traditional preparations may vary in plant material, preparation method, concentration of bioactive constituents, pH, osmolality, sterility, and storage conditions, making their safety difficult to assess based on ethnomedicinal documentation alone. Therefore, the taxa identified in this study should be considered ethnomedicinally relevant priorities for further investigation rather than validated or recommended ophthalmological treatments. Future research should evaluate ocular tolerability and toxicity, formulation stability, and dose–response relationships before any potential clinical application is considered.

Finally, the documentary nature of the study represents an additional methodological limitation. The ethnomedicinal records were not originally generated using standardized data-collection instruments, and consequently the level of detail and consistency of the information varied among sources. The use of predefined search terms and eligibility criteria, independent review, and consensus-based resolution was intended to minimize subjective interpretation during the documentary analysis. The findings, therefore, reflect the information available in the selected repositories and do not necessarily encompass all Mexican ethnomedicinal knowledge concerning the traditional use of plants for ocular conditions.

## 5. Conclusions

The present study provides an expanded ethnopharmacological assessment of medicinal plants traditionally used in Mexico for ocular disorders, documenting 232 ethnomedicinal records corresponding to 174 species, 124 genera, and 57 botanical families. Asteraceae was the most represented family, while *Matricaria chamomilla* was the most frequently cited species. These findings highlight the considerable diversity of plant-based traditional knowledge associated with ocular conditions in Mexico.

A major contribution of this work is the identification of 165 records corresponding to 142 species that were not reported in recent previous publications. These records demonstrate that substantial ethnomedical information remains dispersed across historical, institutional, and digital repositories and is therefore vulnerable to remaining inaccessible to contemporary research.

The integration of ethnomedicinal information with available pharmacological evidence identifies several species of interest for further natural-products and ophthalmological research, while simultaneously revealing important gaps in ocular-specific pharmacology, toxicology, chemical standardization, pharmaceutical formulation, and clinical validation. Importantly, the ethnomedicinal records identified in this study may considered a basis for prioritizing selected species for subsequent pharmacological and toxicological investigation.

The findings also underscore the value of historical and institutional ethnomedical repositories for recovering information that may complement contemporary ethnobotanical literature. The digitization and the systematic disease-oriented indexing of collections such as the IMSS Medicinal Herbarium, together with the continued analysis of historical ethnomedical sources, could substantially improve the accessibility and integration of Mexican traditional medicinal knowledge with contemporary phytochemical, pharmacological and pharmaceutical research.

Overall, this study provides a documented foundation for further investigation of Mexican medicinal plants used traditionally for ocular diseases. However, substantial experimental and clinical research remains necessary to determine whether any of the identified species of their derived products possess reproducible, safe, and clinically relevant ophthalmological effects.

## 6. Future Research Directions

Future research should prioritize species for which ethnomedicinal relevance converges with pharmacological evidence, while also investigating highly cited but poorly validated taxa. Importantly, the experimental evidence specifically addressing ocular pharmacological activity remains limited, and pharmacological effects reported in non-ocular systems should not be interpreted as evidence of efficacy in specific ocular diseases. Therefore, the studies on *Argemone ochroleuca* and members of Fabaceae should include rigorous botanical authentication, chemical characterization of extracts, identification of bioactive constituents, and validation in disease-relevant ocular models. Such studies should incorporate dose–response analyses, mechanisms of action, and comprehensive ocular toxicity assessment. Similarly, antimicrobial activity demonstrated against non-ocular bacterial strains should be further evaluated against clinically relevant ocular pathogens to determine whether such activity is reproducible and potentially relevant to specific ocular infections.

Particular attention should be given to standardization and preclinical evaluation of preparations intended for ocular administration. Standardization of extraction procedures, physicochemical characterization, sterility, stability, ocular tolerability, and reproducibility will be essential before traditional preparations can be considered for further pharmaceutical development. In addition, studies addressing formulation, ocular tissue exposure, and, where appropriate, pharmacokinetic and pharmacodynamic characteristics will be important for determining whether bioactive constituents can reach relevant ocular tissues at effective and safe concentrations.

The systematic digitization of the IMSS Medicinal Herbarium and the development of searchable, disease-indexed ethnomedical databases should also be considered research priorities. Such resources would facilitate integration of traditional knowledge with phytochemical and pharmacological databases and improve the accessibility of Mexican ethnomedicinal knowledge to the international scientific community.

Finally, multidisciplinary collaboration among ethnobotanists, taxonomists, pharmacologists, toxicologists and ophthalmologists will be essential to translate ethnomedicinal observations into reproducible evidence and to identify taxa that merits further pharmaceutical investigation. In this context, the identification of species with traditional ocular uses may be regarded as a starting point for research. The limited availability of experimental studies using appropriate ocular models represents an important research gap and provides a clear rationale for prioritizing rigorous pharmacological, toxicological, and formulation studies in future investigations.

## Figures and Tables

**Table 1:** Mexican medicinal flora for the treatment of eye diseases.

Botanical Family	Scientific Name	Popular Name [English, Spanish or (*Indigenous Language*)]	Disease	Part of the Plant Used	Ethnomedical Preparation	Locality	Reference
Actinidaceae	*Saurauia leucocarpa* Schltdl.	Pipicho	Stinging eyes	Buds	Soak in water, local eye wash	Quimixtlán, Puebla	[14]
Amaranthaceae	*Alternanthera repens* (L.) Kuntze	Tianguispepetla, *Nxiga (otomí)*	Eye irritation	Whole plant	Boiled, in eyes by drops	San Andrés Timilipan, Estado de México	[14]
Anacardiaceae	*Schinus molle* L.	Pirul, árbol de Perú, pirú, Perú. *Peloncuáhuitl*, *copalcuáhuitl*	Spots on the cornea	Leaves, branches and resin	The juice from the branches is applied directly to the eyes daily. The gum is mixed with water to form an emulsion, which is applied directly to the eyes as a cold compress	Ciudad de México	[18]
Anacardiaceae	*Schinus molle* L.	Pepper tree. Pirul, árbol de Perú, bolilla, Perú, pirú. *In the state of Guerrero: preconcuahuitl; in Morelos: copalquahuitl; in Oaxaca: yag lachi (zapoteco); in Puebla: ntaka (popoloca)*	Conjunctivitis, to avoid cataracts. Spots on the cornea	Leaves and fruits	Soak in water, local eye wash. When spots on the cornea, the leaves and fruits are used mixed	Not specified	[17]
Anacardiaceae	*Spondias mombin* L.	Jobo, ciruela, ciruela amarilla, ciruela campechana, ciruelo, ciruelo de hueso grande, ciruelo rojo cimarrón, fondura. *In the state of Nayarit: cua-puat, cuas-pua (cora); in Oaxaca: maxpy, obo (mixe); in Puebla: catan (tepehua), cuauhxonat (nahua), scatan, shipa (totonaco)*	Eye antiseptic	Bark and leaves	Leave to dry in the sun, grind, add boiled water plus a little sea salt. Apply in the eyes by drops	Not specified	[17]
Annonaceae	*Annona cherimola* Miller	Chirimoya	Eye pain	Not specified	Not specified	Not specified	[17]
Apiaceae	*Apium graveolens* L.	Celery. Apio	Eye inflammation	Not specified	Not specified	Not specified	[17]
Apiaceae	*Daucus carota* L.	Carrot. Zanahoria	Tired eyes	Root	Drink the juice	Michoacán	[17]
Apiaceae	*Foeniculum vulgare* (L.) Mill.	Fennel. Hinojo, eneldo, henojo. *In the state of Michoacán: ikiétapu (purépecha); in Puebla: diojo xuitl (náhuatl)*	Eye problems	Not specified	Not specified	Not specified	[17]
Apiaceae	*Foeniculum vulgare* (L.) Mill.	Fennel. Hinojo	Styes, rheumy eyes	Leaves	Wash the eyes daily and several times a day with the water in which the leaves were boiled for 10 min	Ciudad de México	[18]
Apiaceae	*Petroselinum crispum* (Mill.) Fuss	Parsley. Perejil. *In the state of Puebla: perijil, periji*	Conjunctivitis	Not specified	Not specified	Not specified	[17]
Apocynaceae	*Catharanthus roseus* (L.) G.Don	Vicaria, flor de vicaria, micaria, micorro, mulata, ninfa. *In the state of Yucatán: xmicaria (maya)*	Fleshy growth of abnormal tissue of the conjunctiva, corneal opacity, strengthening eyesight	Not specified	Not specified	Not specified	[17]
Apocynaceae	*Mandevilla hypoleuca* (Benth.) Pichon	Flor de San Juan, San Juan, hierba de San Juan, rosa de San Juan, San Juanita. *In the state of Nayarit: nashi*	Eye irritation	Whole plant	Boiled. Daily washes	Not specified	[17]
Apocynaceae	*Plumeria rubra* L.	Cacalosuchil, cacaloxochitl, candelerillo, flor de cuervo, flor de mayo, flor de mayo roja, palo de cacalosúchil, palo de oído, jacalosuchil. *In the state of Guerrero: kakaluxochilt; in Hidalgo: cacaloxuchuil; in Michoacán: parandechicua*	Eye inflammation in children	Root	Not specified	Not specified	[17]
Apocynaceae	*Vallesia glabra* (Cav.) Link	Citabaro, huevito, palo verde. *In the state of Sinaloa: cacarahua; in Sonora: otatabe.*	Eye inflammation, fleshy growth of abnormal tissue of the conjunctiva	Fruits and leaves	Not specified	Sonora	[17]
Apocynaceae	*Vallesia glabra* (Cav.) Link	Cacaragua	Eye inflammation	Latex	Not specified	Culiacán, Sinaloa	[14]
Araceae	*Syngonium podophyllum* Schott	Chapis, chapiso, plátano de ardilla, teléfono. *In the state of Puebla: mudsa mina (otomí), ajahuini, xapis; in San Luis Potosí: kuat (tenek)*	Cataracts, corneal opacity	Leaves	Rubbing	Hidalgo	[17]
Arecaceae	*Brahea dulcis* (Kunth) Mart.	Palma abanico. *In the state of San Luis Potosí: etsol (tenek)*	Cleaning the eyes of a postpartum infant	Stem	Dry the plant in the sun, then boil it in water. Apply drops of the water to the child’s eyes	Guerrero	[17]
Arecaceae	*Cocos nucifera* L.	Coconut. Coco. Cocotero, marón, palma, palma cocotero, palma de coco. *In Ciudad de México: coyolli; in Morelos: coco-coyolli; in Oaxaca: tzon te x a t oan; in San Luis Potosí: lab map, paktda map (tenek)*	Eye inflammation	Not specified	Not specified	Not specified	[17]
Aristolochiaceae	*Aristolochia monticola* Brandegee	Hierba del indio	Rheumy eyes	Not specified	Not specified	Not specified	[17]
Asclepiadaceae	*Asclepias curassavica* L.	Adelfilla, burladora, calderona, cancerillo, cerillo, chilillo, chilillo venenoso, cinco llagas, cojón de gato, cominos rústicos, contrayerba, cresta de gallo, flor de tigre, hierba de la culebra, hierba del sapo, hierba María, hoja delgada, la señorita, Pablito, pericón, ponchilhuite, ponchiuis, revienta muelas, rompe muelas, saca espinas, salvilla, San Pablillo, Santa Rosa, señorita, soldaditos, soldadillo, solimán, venenillo, víbora, viborona. *In the state of Chiapas: pameyat, pameyat warnal, tzajal-chú momol (tzeltal), spama yat, xpamal yat, yich vakax (tzotzil); in Nayarit: temuy (cora); in Oaxaca: ita ya a, yuk paxapaa, yuku xatu*	Eye diseases	Not specified	Not specified	Not specified	[17]
Asclepiadaceae	*Asclepias linaria* Cav.	Algodoncillo, chiche de burra, chichivilla cimarrona, cinco negritos, cola de gato, guajito, hierba del cuervo, patito, pinillo, plumilla, oreja de liebre, solimán, talayote de coyote. *In the state of Michoacán: atu’sh-jiuátsi, chucuipe, iumu turhipiti xukurhi*	Eye diseases	Not specified	Not specified	Not specified	[17]
	*Agave americana* L.	Maguey	Eye inflammation	Root	Root juice	Not specified	[17]
Asphodelaceae	*Aloe vera* (L.) Burm. f.	Sábila	Eye washer	Leaf gel	Crushed	Oaxaca	[17]
Asteraceae	*Artemisia ludoviciana* subsp. *mexicana* (Spreng.) D.D.Keck	Estafiate	Eyes problems	Branches	External. Waving the herbs around the eye and face	Pahuatlán, Puebla	[14]
Asteraceae	*Barkleyanthus salicifolius* (Kunth) Rob. & Brett.	Faja de algodón, faja de borrego. Azomiate, azumiate, camiso macho, chilca, flor de dolores, higuerilla, jara, jaral, jara mexicana, jarilla amarilla, jarilla blanca, jarilla verde, jaralillo, pajarilla. *Izcatzoyatl (náhuatl)*, *Necheloli (náhuatl). In the state of Estado de México: necheloli (náhuatl), ra-yhotitha (otomí), yescha (mazahua); in Michoacán: it oksini (purhépecha); in Morelos: izcatzóyatl (náhuatl), alzumihatl; in Oaxaca: baldag shi ingol (zapoteco)*	Eyes problems	Not specified	Not specified	Not specified	[17]
Asteraceae	*Barkleyanthus salicifolius* (Kunth) Rob. & Brett.	Azumiate	Conjunctivitis, corneal opacity	Whole plant	Boil the branch in a cup of water and left overnight. Use the resulting liquid to wash the eyes before bed. When the eyes are slightly inflamed, apply the leaves directly to your eyelids. Smudge for healing the eyes	Izúcar de Matamoros, Puebla	[18]
Asteraceae	*Calendula officinalis* L.	Mercadela, alta reyna, caléndula, mercadel	Conjunctivitis	Not specified	Not specified	Not specified	[17]
Asteraceae	*Calendula officinalis* L.	Mercadela	Conjunctivitis	Flowers	Boiled. Wash the eyes with the warm liquid	Ciudad de México	[18]
Asteraceae	*Cirsium ehrenbergii* Sch. Bip.	Cardo	Eye irritation	Leaves	Not specified	Not specified	[17]
Asteraceae	*Cirsium mexicanum* DC.	Cardosanto, carlosanto	Red eyes, cataracts, poor vision	Not specified	Juice. Smear on the eyes	Not specified	[17]
Asteraceae	*Helianthus annuus* L.	Girasol	Eye pain	Not specified	Not specified	Not specified	[17]
Asteraceae	*Heterotheca inuloides* Cass.	Árnica. Árnica de campo, árnica de monte, árnica del país, cuateteco, cuauteteco, falsa árnica, hornilla, tabaco de las montañas. *In the state of Estado de México: reemn, riemn (mazahua); in Guerrero: acahual, akawtomitl; in Michoacán: árnica (purépecha)*	Eye washer	Asteraceae	*Heterotheca inuloides* Cass.	Not specified	[17]
Asteraceae	*Laennecia filaginoides* DC.	Simonillo, falso sacalechinchi, gordolobo, hierba de las ánimas, rosilla, simonío, *zacachichíc*	Weak eyes, cataracts	Not specified	Juice	Not specified	[17]
Asteraceae	*Matricaria chamomilla* L.	Chamomile. Manzanilla	Eye irritation	Leaves and flowers	Boiled	Xochimilco, Ciudad de México	[14]
Asteraceae	*Matricaria chamomilla* L.	Chamomile. Manzanilla	Conjunctivitis	Flowers	Infusion. Eye washer	Curijo, Estado de México	[14]
Asteraceae	*Matricaria chamomilla* L.	Chamomile. Manzanilla	Eye irritation	Leaves	Boiled	Quimixtlán, Puebla	[14]
Asteraceae	*Matricaria chamomilla* L.	Chamomile. Manzanilla	Eye irritation	Stems	Boiled. Drops in the eye	Juan Aldama, Zacatecas	[14]
Asteraceae	*Matricaria chamomilla* L.	Chamomile. Manzanilla	Conjunctivitis, styes, eye irritation	Flowers	Boiled. Wash the eyes. Apply warm or hot compresses for 15 min	Ciudad de México	[18]
Asteraceae	*Matricaria chamomilla* L.	Chamomile. Manzanilla, camomila. *In the state of* *Michoacán: amantsania* *(purépecha)*	Eye problems, eye inflammation, eye irritation, rheumy eyes, eye pain, to wash eyes	Not specified	Not specified	Not specified	[17]
Asteraceae	*Matricaria chamomilla* L.	Chamomile. Manzanilla	Eye pain	Branches	Boiled. Wash the eyes	Misantla, Veracruz	[14]
Asteraceae	*Matricaria chamomilla* L.	Chamomile. Manzanilla	Rheumy eyes	Not specified	Not specified	Pátzcuaro, Erongarícuaro, Michoacán	[14]
Asteraceae	*Matricaria chamomilla* L.	Chamomile. Manzanilla. *In the state of Chiapas: manzanoica; in Oaxaca: queza (zapoteco)*	Eye irritation, conjunctivitis, styes, inflammation of tear ducts	Flowers	For the treatment of conjunctivitis and eye irritation, the flowers are boiled and left overnight. The following day, the infusion is applied as drops to the eyes. For styes, the eyes are washed several times a day. For inflammation of the tear ducts, warm compresses of the flower infusion are placed on the eyes for 15 min	The whole country (México)	[16]
Asteraceae	*Parthenium hysterophorus* L.	Escobilla o hierba de la hormiga, hierba de la hormiga, altamisa, altanisa, canario, claudiosa blanca, confitillo, escoba amargosa, escobilla, hierba amargosa. *In the state of Oaxaca: arternis ujts; in Puebla: ix ilthin kgolhnu (totonaco), sumia papalsni (tepehua); in Quintana Roo: jaway, x-huaway (maya)*	Rheumy eyes	Not specified	Not specified	Not specified	[17]
Asteraceae	*Sanvitalia procumbens* Lam.	Ojo de gallo. Ojo de chanate, ojo de gallina, ojo de gato, ojo de perico, ojo de pollo. *In the state of Morelos: coyoixtli (náhuatl); in Yucatán: yanix k’ aantun buuba, k’aan tun buub, xk’antun buub (maya)*	Eye problems	Not specified	Not specified	Not specified	[17]
Asteraceae	*Sonchus oleraceus* L.	Lechuguilla. Achicoria, chicoria, diente de león, endivia, hierba del golpe, matlali morado. *In the state of Estado de México: yemancatotol quilitl; in Michoacán: tlamatsalin; in San Luis Potosí: ejtil malil koy, cholich toro (tenek)*	Opacity	Not specified	Not specified	Not specified	[17]
Asteraceae	*Tagetes erecta* L.	Cempasúchil, flor de muerto, flor de veinte pétalos; Cepazuchilt, cempasochil, cempoal, flor de cempoal, flor de cempoaxochitl, flor de difunto, flor de muerto chino, flor de niño, veinte flores; *in the state of Ciudad de México: cempoalxochitl (náhuatl); in Estado de México: tecacaya, zempoalxochitl; in Guerrero: yita cua (mixteco)*	Rheumy eyes, weak eyes, opacity, leukoma	Flowers	Flowers mixed with honey		[17]
Asteraceae	*Tagetes lucida* Cav.	Pericón, anisillo, atagote, cedrón, flor de xuchitl, hierbanís, hierba anís, hierba de nubes, hierba de San Juan, hierba santa, periquillo, rincón, Santa María, Santa María de jardín, tatalencho, yerbanís. *In the state of Chiapas: k’anal nich wamal, k’ixin vomol, perikon vomol, tzitz ak, tzitz pox, tzitz vomol, tzitzilal ul (tzotzil), tzitzak (tzeltal); in Estado de México: mikua (otomí), xonequilitl; in Guerrero: yita perico (mixteco), cuahuyahuitli; curucume*	Opacity	Not specified	Not specified	Not specified	[17]
Asteraceae	*Tanacetum parthenium* (L.) Sch.Bip.	Hierba de Santa María	Eye irritation	Leaves	Decoction, wash the eyes	Misantla, Veracruz	[14]
Asteraceae	*Tanacetum parthenium* (L.) Sch.Bip.	Santa María	Conjunctivitis, rheumy eyes	Leaves	The leaves are rubbed directly on the eyelids. It is common to feel a strong burning sensation	Ciudad de México	[18]
Asteraceae	*Taraxacum officinale* Weber ex F.H. Wigg.	Dandelion. Diente de león, amargón, borraja, cerraja, chicoria, chinita, lechuguilla. *In the state of Estado de México: cuanacaquilitl (náhuatl); in Michoacán: uirapen kauerin (purhepecha); in Oaxaca: ixpule*	Eye irritation	Fresh sap or dried plant	Apply fresh sap or the water in which the dried plant has been soaked	Not specified	[17]
Asteraceae	*Verbesina crocata* (Cav.) Less.	Capitaneja, árnica capitaneja, capitaneja anaranjada, palo espino. *In the state of Guerrero: cachanquillo; in Morelos: chimalacatl; in Yucatán: ki’che, taamkas che’*	Eye inflammation, eye problems	Not specified	Not specified	Not specified	[17]
Asteraceae	*Verbesina crocata* (Cav.) Less.	Capitaneja, cuatro dorsos. *Nahuitiput*, *nahuitépuz*	Blepharitis	Root	The dried roots are cut and placed in boiling water for 10 min. The sick person drinks 1/4 cup daily of the liquid	Ciudad de México, Puebla, Hidalgo y Veracruz	[18]
Asteraceae	*Verbesina pedunculosa* (DC.) B.L.Rob.	Capitaneja. *In the state of Morelos: chimalacatl*	Eye problems	Not specified	Not specified	Not specified	[17]
Asteraceae	*Verbesina persicifolia* DC.	Huichin. *In the state of Veracruz: taxiwua; in Tlaxcala: tlamacas*	Red eyes	Not specified	Not specified	Not specified	[17]
Begoniaceae	*Begonia gracilis* Kunth	Chipile, orejita de guajolote, alita de ángel	Eye inflammation	Root	Crushed in liquid	Not specified	[17]
Berberidaceae	*Berberis moranensis* Hebenstr. & Ludw. ex Schult. & Schult.f.	Palo de muerto. *In the state of Estado de México: huitzcolotl; in Hidalgo: xoxoco.*	Eye irritation	Branches, bark	Decoction. The peel is scraped from the stems and placed in a glass with water and left overnight. Next morning the eyes are washed, then it is done every third day	Hidalgo	[17]
Brassicaceae	*Brassica oleracea* L.	Repollo	Eye inflammation	Not specified	Not specified	Michoacán	[17]
Brassicaceae	*Brassica rapa* subsp. *oleifera* (DC.) Metzg.	Nabo	Eye problems	Not specified	Not specified	Huitel, Hidalgo	[14]
Cactaceae	*Opuntia cochenillifera* (L.) Mill.	Nopal. *In the state of San Luis Potosí: pak’ak’ (tenek)*	Eye problems	Not specified	Not specified	Not specified	[17]
Campanulaceae	*Lobelia laxiflora* Kunth	*Bixtoní (otomí)*	Eye infection	Latex	Not specified	San Pablito, Pahuatán, Puebla	[14]
Campanulaceae	*Lobelia laxiflora* Kunth	Chilpanxochitl, aretitos, calzón de don Juan, campanitas, contrahiedra, flor tocada, santa quiteria. *In the state of Ciudad de México: acaxóchitl; in Michoacán: amamba huemberekua, vamberakua (purhépecha), in Puebla: bixtoni, tonzutli (otomí). Tlacualborrego (náhuatl)*	Eyes problems	Latex	Not specified	Not specified	[17]
Campanulaceae	*Lobelia laxiflora* Kunth	Flor tocada	Eye infection	Latex	Not specified	San Pablito, Pahuatán, Puebla	[14]
Celastraceae	*Maytenus phyllanthoides* Benth.	Mangle dulce	Cataracts	Wood	Infusion of the wood	Not specified	[17]
Cistaceae	*Crocanthemum glomeratum* (Lag.) Janch.	Cenicilla, hierba de la gallina, hoja ceniza. cenicillo, cenicillo amarillo, cenicillo negro, cilantrillo de monte, damiana, flor del golpe, hierba ceniza, hierba del moro, Juana-Juana, Juanita, palo cenizo, Santa Martha, tacato, tomillo de monte. *Nehnexticxihuitl (náhuatl)*	Tired eyes, corneal opacity, leukoma	Flowers	Mixed flowers with honey	Not specified	[17]
Combretaceae	*Combretum farinosum* Kunth	Bejuco de carape, Carape, escobetillo, guam viejo, peineta, peinetillo.	Eye infection, Corneal opacity.	Sap	Not specified	Michoacán	[17]
Commelinaceae	*Commelina erecta* L.	Hierba del pollo, matlali. atlalina, tripa de gallina. *In the state of Puebla: atlic (náhuatl), kasmalj, malastis; in Quintana Roo: kabalsit, pahtsa’, xbubulha’, ya’axha’ (maya); in Yucatán: kabalsit, ko’lebil, pah-tsa’, x-habul-ha, ya’ax-ha-xiu*	Eyes problems, conjunctivitis, eyes infection, tired eyes	Inflorescence	Directly to the eyes	Quintana Roo	[17]
Commelinaceae	*Commelina tuberosa* L.	Hierba del pollo, chipil de piedra, matlalina, pata de pollo, tripa de pollo, tripa de pollito. *In the state of Veracruz: chic-ze*	Eye problems	Not specified	Not specified	Not specified	[17]
Convolvulaceae	*Ipomoea stans* Cav.	Galuza	Eye inflammation	Not specified	Not specified	Not specified	[17]
Convolvulaceae	*Turbina corymbosa* (L.) Raf.	Angelito o bejuco de San Pedro, bejuco de San Pedro, flor de Pascua, hoja del norte, Santa Catarina, semillas de la virgen o hierba María (Oaxaca). *In the state of Puebla: sachxoit (tepehua); in Quintana Roo: tabentun, xtabentun*	Eye diseases	Seed	Ground with milk and chili and smeared on the head and forehead	Not specified	[17]
Costaceae	*Costus spicatus* (Jacq.) Sw.	Caña de jabalí. Bordón de viejo, caña de venado, cañita agria, cañita de monte, vara de San José. *In the state of Puebla: ix chan kat paxni (tepehua), ixkayonto (otomí), ixtanchakgt paxni (totonaco)*	Eye infection	Young stem	Squeeze the stem and apply drops to the eye	Not specified	[17]
Crassulaceae	*Kalanchoe pinnata* (Lam.) Pers.	Siempreviva, tronador. Admirable, amor, belladona, bolsita, bruja, flor de arete, flor fresca, hierba maravillosa, hierba de la lechuza, hoja fresca, hoja vidriosa, maravilla, pericón, sánalo todo, San Juan, San Nicolás. *In the state of Chiapas: anuktik siquel vomol; Oaxaca: tods ujts, toxk ujts; Puebla: akaxukuch, huey atlic (náhuatl), joxi patei (otomí); in San Luis Potosí: tolow xekel, lam ts’ojol (tenek)*	Eye washer with the juice of the leaves. Apply 3 or 4 drops of the juice in each eye	Leaves	Juice of the leaves	Not specified	[17]
Crassulaceae	*Sedum dendroideum* Moc. & Sessé ex DC.	Siempreviva	Fleshy growth of abnormal tissue of the conjunctiva	Leaves	Rubbed	San Luis Teolocholco, Tlaxcala	[14]
Crassulaceae	*Sedum dendroideum* Moc. & Sessé ex DC.	Siempreviva, flor de siempreviva, siempre viva amarilla	Cataracts, corneal opacity, fleshy growth of abnormal tissue of the conjunctiva	Leaves	The juice of the leaves is applied directly to the eyes	The whole country (Mexico)	[16]
Crassulaceae	*Sedum dendroideum* Moc. & Sessé ex DC.	Siempreviva	Eye problems	Leaves	Not specified	San Dieguito, Estado de México	[14]
Crassulaceae	*Sedum dendroideum* Moc. & Sessé ex DC.	Siempreviva. *Tetzmitl*	Cataracts, corneal opacity, fleshy growth of abnormal tissue of the conjunctiva, eye irritation	Leaves, juice	The leaves are squeezed to obtain the juice, which is placed directly in the eyes: 3 drops in the morning and 3 drops at night. It burns	Ciudad de México, Hidalgo, Morelos, Puebla	[18]
Crassulaceae	*Sedum dendroideum* Moc. & Sessé ex DC.	Siempreviva, siempreviva gruesa. *In the state of Estado de México: medye (mazahua); in Puebla: kanda chansa (popoloca)*	Corneal opacity, fleshy growth of abnormal tissue of the conjunctiva, eye irritation, eye problems	Not specified	Not specified	Not specified	[17]
Crassulaceae	*Sedum praealtum* A.DC.	Siempreviva	Eye irritation,	Leaves	Squeeze the leaves directly into the eyes	Not specified	[17]
Cucurbitaceae	*Cucurbita ficifolia* Bouché	Calabacilla. *In the state of San Luis Potosí: tduk’uk’,tdakpen tduk’uk’ (tenek)*	Eye inflammation	Not specified	Not specified	Not specified	[17]
Cucurbitaceae	*Cucurbita maxima* Duchesne	Calabaza, melón	Eye inflammation	Not specified	Not specified	Not specified	[17]
Cucurbitaceae	*Momordica charantia* L.	Cundeamor, amor seco, bálsamo, bejuco condeamor, chorizo, cochinito, cosquelite, flor de amor, manzanina. *In the state of Oaxaca: pa xandia (mixe); in Quintana Roo: kolmol, lol-much (maya); in Yucatán: kool mol, xcochinito, xkool mool, yakunaj aak’, yaa kunaj aak’ (maya)*	Eye inflammation	Not specified	Not specified	Not specified	[17]
Euphorbiaceae	*Cnidoscolus multilobus* (Pax.) I.M. Johnst.	Mala mujer, mala mujer de cerro, ortiga. *In the state of Oaxaca: chench tiek; in Puebla: cajni (tepehua), gahni, kgajna, kakne (totonaco), tectzon-quilit (nahua), tzitzitcastle (náhuatl); in Veracruz: kakne (totonaco); in San Luis Potosí: ak’ (tenek)*	Eye problems	Not specified	Not specified	Not specified	[17]
Euphorbiaceae	*Croton ciliatoglandulifer* Ortega	Duraznillo, mala mujer, ciega vista, copalito, dominguilla, hierba mala, hoja sierra, palo duraznillo, picosa, solimán. *In the state of Guerrero: tonalocote; in Oaxaca: neajmier sox. xonaxe; in Puebla: teclate; in Sonora: uruquenia*	Eye infection	Not specified	Not specified	Not specified	[17]
Euphorbiaceae	*Croton cortesianus* Kunth	Not specified	Eye infection	Sap	Not specified	Not specified	[14]
Euphorbiaceae	*Croton draco* Schltdl.	Sangregrado, angregado, grado, palo muela, palo de sangre de drago, palo sangriento, piñón, sangre de draco, sangre de drago, sangre de grado, sangre de perro, sangre de toro. *In the state of Chiapas: ch’ich bat (tzeltal), ch’ich bot (tzotzil), chichbot, chchie, chucum; in Oaxaca: ma min; in Puebla*: *yescuitl (náhuatl)*	Eye infection	Not specified	Not specified	Not specified	[17]
Euphorbiaceae	*Croton niveus* Jacq.	Copalchi. *In the state of Oaxaca: copalchin; in Yucatán: chul, chul che’, ch’uju che’, kopal che’; pomolche’*	Eye problems	Not specified	Not specified	Not specified	[17]
Euphorbiaceae	*Croton reflexifolius* Kunth	Not specified	Eye problems	Resin	Not specified	Not specified	[14]
Euphorbiaceae	*Croton repens* Schtdl.	Sangre de grado de la sabana. *Soj kobak*, *soj muk (Popoluca)*	Retinal problems	Sap	Plant sap is applied to the eye to cleanse the retina	Not specified	[14]
Euphorbiaceae	*Croton soliman* Cham. & Schltdl.	*P’ere’esk’uuch (maya)*	Eye infection	Latex	Not specified	Yaxcabá, Yucatán	[14]
Euphorbiaceae	*Euphorbia anychioides* Boiss.	Hierba de la golondrina	Eye irritation, expulsion spines from the eyes	Whole plant	Wash the eyes with the water leave overnight of the boiled whole plant and rosebuds	Not specified	[17]
Euphorbiaceae	*Euphorbia heterophylla* L.	Picachali, quebranto. *In the state of Oaxaca: ndiichtog amarillo, ndiichtog maril; in Puebla: tiriciaxihuitl; in Quintana Roo: juboncac (maya); in Yucatán: chuleb xiw, jobon k’aak’, yanajobonte’ k’aak’, xtop’lan xiix*	Eye problems	Latex	Not specified	Not specified	[17]
Euphorbiaceae	*Euphorbia hirta* L.	Hierba de la golondrina	Conjunctivitis	Not specified	Not specified	Hidalgo	[14]
Euphorbiaceae	*Euphorbia hirta* L.	Golondrina	Fleshy growth of abnormal tissue of the conjunctiva	Whole plant	Wash the eye with the water of the boiled plant	Not specified	[14]
Euphorbiaceae	*Euphorbia hypericifolia* L.	Golondrina	Styes	Latex	Drops	San Pablito, Pahuatán, Puebla	[14]
Euphorbiaceae	*Euphorbia maculata* L.	Hierba de la golondrina. *In the state of Hidalgo: tlalmemeya*	Corneal opacity, eye discharge	Not specified	Not specified	Not specified	[17]
Euphorbiaceae	*Euphorbia maculata* L.	Hierba de la golondrina	Corneal opacity	Whole plant	Crush the plant until you obtain a pulp. Squeeze out this pulp and place 2 or 3 drops on the eyes, 3 times a day until healed	Ciudad de México, Sonora, Durango, San Luis Potosí, Jalisco, Michoacán, Estado de México, Tlaxcala, Puebla and Veracruz	[18]
Euphorbiaceae	*Euphorbia prostrata* Aiton	Hierba de la golondrina, golondrina, hierba de la golondrina chica, hierba del soldado. *In the state of Michoacán: uijsakua-pitsikat; in Nayarit: santaru (cora); in Oaxaca: mejen xanab mucuy, xanab—mucuy; in Puebla: akgkunuc xanat (totonaco)*	Eye problems, corneal opacity, eye discharge	Leaves	Squeeze over the eyes until feel relief	Sonora, Nayarit, Ciudad de México, Michoacán, Puebla, Tlaxcala	[17]
Euphorbiaceae	*Euphorbia prostrata* Aiton	Hierba de la golondrina, golondrina blanca, hierba de la golondrina chica. *In the state of Puebla: akgkunuc xanat (totonaco)*	Corneal opacity, cataracts	Leaves, stems	A paste is made from the leaves and stems to extract the milky juice, and 1 or 2 drops are placed on the affected eye	Ciudad de México, Estado de México, Guanajuato, Hidalgo, Michoacán, Puebla, Sonora	[16]
Euphorbiaceae	*Euphorbia thymifolia* L.	Hierba de la golondrina	Eye problems	Latex	Drops	Ingenio Dos Patrias, Tabasco	[14]
Euphorbiaceae	*Euphorbia* sp.	Tlanechtzi	Fleshy growth of abnormal tissue of the conjunctiva	Leaves	Wash the eye with the water of the boiled plant	Tlacotepec de Díaz, Puebla	[14]
Euphorbiaceae	*Jatropha curcas* L.	Piñón, palo de piñón, piñón bronco, piñoncillo. *In the state of Morelos: cuauixtli (náhuatl); in Oaxaca: kutsekeey, tzon texoa kichi; in Puebla: axti, cahuax (nahua), chu.ta (totonaco), taktinau (tepehua), temuza (otomí)*	Eye inflammation, eye discharge, leukoma	Not specified	Not specified	Not specified	[17]
Euphorbiaceae	*Jatropha dioica* Seseé	Sangre de drago, sangregado. sangre de grado, sangregrada	Eye irritation, corneal opacity, to cure blindness	Fruit	Squeeze the fruit over the eyes, letting two or three drops fall	Not specified	[17]
Fabaceae	*Caesalpinia pulcherrima* (L.) Sw.	Barbona, cabello de ángel, camarón, espuela de Santiago, flor de camarón, framboyán, maravillo, rosal amarillo, rosal rojo, tabaquín; *Chacaloxóchitl*, *xiloxóchitl (náhuatl). In the state of Estado de México: ciringuanico, sexinguanico; in Michoacán: siringuanico, (purepecha), surundanico; in Puebla: sutusani’, sutusoníl (totonaco)*	Eye problems	Not specified	Not specified	Not specified	[17]
Fabaceae	*Calliandra grandiflora* (L’Hér.) Benth.	Cabello de ángel; cabellitos de ángel, cabellos de ángel, lele, pambonato, tabardillo, timbre, timbrillo. *In the state of Ciudad de México: tzonxochitl; in Guerero: tlacoxiloxóchitl; in Puebla: saqaqa (totonaco)*	Eye problems	Not specified	Not specified	Not specified	[17]
Fabaceae	*Calliandra houstoniana* Standley	Cabello de ángel, tabardillo, timbre, timbrillo rojo. *In the state of Puebla: tzotzon (totonaco), tzontoto yacuahuit; in QuintanaRoo: k’asin, k’anlsin (maya); in San Luis Potosi: wit ’oot (tenek)*	Conjunctivitis	Flower stamens	Wash the eyes with the infusion	Puebla	[17]
Fabaceae	*Entada polystachya* (L.) DC.	Bejuco prieto. *In the state of Estado de México: bejuco de paringue, paringue; in Oaxaca: yei, yey*	Eye infection	Stem	Not specified	Not specified	[17]
Fabaceae	*Erythrina flabelliformis* Kearney	Chilicote	Eye pain, improving eyesight	Not specified	Not specified	Not specified	[17]
Fabaceae	*Eysenhardtia polystachya* (Ortega) Sarg.	Palo dulce, chiquiliche, coatillo, palo cuate, palo dulce, rosilla, taray, vara dulce, varaduz. *In the state of Ciudad de México; ursa (otomí); in Guerrero: yitu bishi (mixteco). coatli; in Nayarit: bisasa (cora); in Oaxaca: ‘ma soo; in San Luis Potosí: chilab te’, tsakam wayal (tenek)*	Eye infection	Not specified	Not specified	Not specified	[17]
Fabaceae	*Gliricidia sepium* (Jacq.) Kunth	Cacahuananche, cocoite, cocuite, madre cacao, matarrata, matarratón, trébol. *In the state of Michoacán: canahuam*; in *Oaxaca: najmbeal, tsooky*; *in Quintana Roo: sak va’ab, sakyab (maya); in Veracruz: paque, p’aqui, uabaxiuit*	Eye pain	Leaves	Wash the eyes with the water used to soak the leaves	Not specified	[17]
Fabaceae	*Medicago sativa* L.	Alfalfa	Improving eyesight	Not specified	Not specified	Not specified	[17]
Fabaceae	*Mimosa albida* Humb. & Bonpl. ex Willd.	Dormilona grande	Blurred vision	Root	Boiled and applied in drops to the eyes	Not specified	[14]
Fabaceae	*Mimosa albida* Humb. & Bonpl. ex Willd.	Vergonzosa, dormilona, dormilona grande, espina dormilona, espinita dormilona, raíz de uña de gato, uña de gato, zarsa sensitiva, zarza viva. *In the state of Guerrero: yucu ndita a (mixteco); in Hidalgo: cochohuistle; in Oaxaca: ’oo tú cu’*	Blurred vision	Root	Boiled and soaked overnight. Apply drops to the eye	Not specified	[17]
Fabaceae	*Neltuma glandulosa* (Torr.) Britton & Rose	Mezquite	Eye irritation, eyelid inflammation	Gum, leaves	For eyelid inflammation, dissolve the gum in warm water and place it in the eye as drops once a day. For eye irritation, apply 5 drops of the leaf juice in the morning	Coahuila, Durango, Michoacán, Morelos, Nuevo León	[16]
Fabaceae	*Neltuma juliflora* (Sw.) Raf.	Mezquite. *Mízquitl*	Eye irritation, Blepharitis	Leaves, gum	For eye irritation, apply 5 drops of the leaf juice directly to the eyes. The gum solution in water is applied as drops to the eyes for blepharitis	Ciudad de México	[18]
Fabaceae	*Neltuma juliflora* (Sw.) Raf.	Mezquite, algarrobo, huizache mexicano, pechita. *In the state of Estado de México: tziritsequa; in Oaxaca: huamelula coyul, mangan patier, patier de burra, patier lam (huave)*	Eye irritation, eye problems	Leaves	Ground leaves in boiled water, strain and apply as drops in the eyes	Nayarit, Sonora, Estado de México, Oaxaca	[17]
Fabaceae	*Neltuma juliflora* (Sw.) Raf.	Mezquite	Eye inflammation	Gum	In water	Not specified	[14]
Fabaceae	*Neltuma laevigata* (Humb. & Bonpl. ex Willd.) Britton & Rose	Copal, copalillo, puam. *In the state of Puebla: pum (totonaco), copalhuijiac; in San Luis Potosí: jom te’ (tenek)*	Eye problems, conjunctivitis	Leaves buds	Ground and strained leaves buds in a clean cloth. Grind the buds with a little breast milk, grill them on a griddle	Morelos	[17]
Fabaceae	*Pediomelum pentaphyllum* (L.) Rydb.	Contra yerba, contra yerba blanca.	Cataracts	Wood	Decoction	Not specified	[17]
Fabaceae	*Phaseolus vulgaris* L.	Frijol, frijol de vara, frijol negro. *In the state of Michoacán: tatsini siuari (purhépecha); in Puebla: baju, et, stapu, yetl; in Veracruz: kastapu (totonaco); in San Luis Potosí: tsanok’, lej tsanuk’ (tenek)*	Corneal opacity, fleshy growth of abnormal tissue of the conjunctiva	Seeds	Roasted and ground seeds are placed on a castor bean stem and blown onto the eye. Single dose	Not specified	[17]
Fabaceae	*Pithecellobium dulce* (Roxb.) Benth.	Guamúchil, guamúchil amargo, guamúchil dulce, guamúchil hogadizo, guamuchilo, huamuchil, palo de guamoche, pinzan. *In the state of Guerrero: tichi coo bíchí, tíchí coo yatu (mixteco); in Hidalgo: humu, umu; in Oaxaca: piil (huave), íson te cho (amuzgo)*	Corneal opacity	Sap	Apply 2 to 3 drops of sap in the morning	Not specified	[17]
Fabaceae	*Vachellia cornigera* (L.) Seigler & Ebinger	Cornezuelo, *tsujpin (totonaca)*	Blurred vision	Young plant	Boiled, for oral administration	Mecapalapa, Pantepec, Puebla	[14]
Fabaceae	*Vachellia cornigera* (L.) Seigler & Ebinger	Cornezuelo	Blurred vision	Whole plant	Boiled. Drink three cups a day	Not specified	[17]
Fabaceae	*Vachellia farnesiana* (L.) Wight & Arn.	Huisache	Corneal opacity, stinging	Not specified	Not specified	Not specified	[17]
Fabaceae	*Vachellia pennatula* (Schltdl. & Cham.) Seigler & Ebinger	Tepame	Eye irritation, eye problems	Cáscara	Soak the peel in water overnight. Apply 2 drops per day to the eyes	Not specified	[17]
Fabaceae	*Vicia faba* L.	Haba. *Da-qu (otomí)*	Red eyes	Leaves	Crushed leaves in water and applied as drops to the eyes	Not specified	[14]
Fabaceae	*Vicia faba* L.	Haba	Eye irritation	Leaves	Powdered	Local	[14]
Fabaceae	*Vicia faba* L.	Haba, haba amarilla, hoja de haba. *In the state of Estado de México: da-ku (otomí); in Michoacán: jaasi (purhépecha), jasi*	Eye irritation	Leaves	The fresh leaf is ground, and the juice is applied to the eyes	Estado de México	[17]
Geraniaceae	*Geranium mexicanum* Kunth	Geranio, geranio de olor, pata de león. *In the state of Estado de México: tlacecehuitl; in Michoacán: pupurhajkura*	Eye inflammation	Not specified	Not specified	Not specified	[17]
Lamiaceae	*Agastache mexicana* (Kunth) Lint & Epling	Toronjil	Cry without tears	Not specified	Not specified	Not specified	[17]
Lamiaceae	*Clinopodium brownei* (Sw.) Kuntze	Escobilla, hierba del aire; *tlachpahuaste (náhuatl)*, *yeca-xuitl (náhuatl)*	Eye problems	Branches	External. Waving the herbs around the eye and face	Xolotla, Pahuatlán, Puebla	[14]
Lamiaceae	*Marrubium vulgare* L.	Marrubio, malcubio, malva del sapo, malvarrubina, marrubio blanco, mata ceniza. *In the state of Baja California Norte: melcupis; in Estado de México: tzopiloshihuitl; in Michoacán: k’ameri, vitsacua, vitzacua*	Eye problems	Not specified	Not specified	Not specified	[17]
Lamiaceae	*Mentha arvensis* L.	Hierba buena, yerbabuena, poleo. *In the state of Michoacán: kuatsitasi (purhépecha), tsukuri; in Puebla: albobueno (nahua), xakandrehe (otomí)*	Eye irritation	Not specified	Not specified	Not specified	[17]
Lamiaceae	*Ocimum basilicum* L.	Albahacar	Fleshy growth of abnormal tissue of the conjunctiva	Seeds	Not specified	Colotmuk, Yucatán	[14]
Lamiaceae	*Ocimum basilicum* L.	Albahacar, albacar, albacar corriente, albacar hembra, albacar macho albahaca, albahaca blanca, albahaca corriente, albahaca de castilla, albahaca de la tierra, albahaca morada, albahaca arribeña, orégano. *In the state of Guerrero: albaka (náhuatl); in Oaxaca: xuuikh, xuuiky; in Puebla: xpasimakatoro; in Quintana Roo: albuja’kar (maya)*	Corneal opacity	Not specified	Not specified	Not specified	[17]
Lamiaceae	*Ocimum campechianum* Mill.	Albahacar, albahacar del monte, albaca, albacar, albacon, albahaca cimarrona, albahaca de hoja ancha, albahaca de tierra, albahaca silvestre, albahacar morada, chiripitillo. *In the state of Oaxaca: ma mi, pa xuuiky; in Quintana Roo: x-cacaltun (maya)*	Clean the eyes, conjunctivitis, cataracts	Seeds	Not specified	Not specified	[17]
Lamiaceae	*Origanum majorana* L.	Mejorana	Eye inflammation	Not specified	Not specified	Not specified	[17]
Lamiaceae	*Prunella vulgaris* L.	Albahacar, cabezona, hierbabuenilla, cola de ratón, hierba del cáncer, hierba del pollo, hierba del puerquito, mirto, olotillo morado, taponcillo. *In the state of Chiapas: alavena tz’i’lel, vojton vomol, yaxal nich vomol (tzotzil), xayal nichim, yaxal nichim wamal (tzeltal)*	Eye infection, eye problems	Not specified	Not specified	Not specified	[17]
Lamiaceae	*Salvia coccinea* Buc’hoz ex Etl.	Tlachinolxóchitl	Eye infection	Not specified	Not specified	Tamazunchale, San Luis Potosí	[14]
Lamiaceae	*Salvia hispanica* L.	Chía, salvia	Eye problems	Seeds	Place the seeds on the eyes and wait for them to swell. This facilitates the expulsion of larvae or any foreign body of the eye	Not specified	[17]
Lamiaceae	*Salvia microphylla* Kunth	Mirto, diente de acamaya, hierba de mirto, mastranzo, mirto chico, mirto cultivado, mirto de Castilla, toronjil, verbena. *In the state of Estado de México: mistro, mistru, simbaregne (mazahua); in Michoacán: mustia (purhépecha); in Puebla: ixtasalak (tepehua), kamirto nchia (popoloca)*	Eye problems	Flowers and juice	Apply gently to the eyelid until healed	Not specified	[17]
Lamiaceae	*Salvia polystachya* Cav.	Chía	Expulsion of foreign body of the eye	Not specified	Not specified	Not specified	[17]
Lamiaceae	*Salvia rosmarinus* Spenn.	Romero. *In Michoacán: romeru*	Eye problems	Not specified	Not specified	Not specified	[17]
Lamiaceae	*Salvia rosmarinus* Spenn.	Romero	Eye irritation, styes	Branches, leaves	The branches are crushed and added to water, then left overnight. The resulting liquid is used to wash the eyes daily before bed. This liquid can be warm	Ciudad de México, Jalisco	[18]
Lamiaceae	*Vitex mollis* Kunth	Coyotomate, capulincillo, nanche de perro. *In the state of Estado de México: cuahuilote, uvalana; in Guerrero: cuyomate. In Morelos: cuyotomate; in Oaxaca: beo*	Eye problems, rheumy eyes, corneal opacity	Buds	The bud is squeezed over the eye	Not specified	[17]
Loganiaceae	*Spigelia* sp.	Pirimón vomol	Conjunctivitis	Sap	Not specified	Chiapas	[14]
Loranthaceae	*Psittacanthus calyculatus* G. Don	Injerto, caballera, chimpilla, corriguela, corrigurla, hoja de opinión, lirio parásito, muérdago, secapalo. *In the state of Morelos: cuaquetzpallaguali (náhuatl); in Oaxaca: ’oo dsi có née (chinanteco); in Puebla: tepalcat (nahua); in Quintana Roo: xcubemba (maya)*	Conjunctivitis	Leaves	Crushed with water. Wash the eyes	Tabasco	[17]
Lythraceae	*Cuphea aequipetala* Cav.	Hierba del cáncer, alcancer, alfilerillo, atlanchana, chachanchillo, cordón de San Francisco guinda, hierba del ángel, hierba del descancer, miel de abeja, mirto, mirto morado, moyote, pelatito, rama roja, topo de indias, tripa de tuza. *Apancholida (náhuatl)*; *tozancuitlacxolli (náhuatl)*	Eye problems	Not specified	Not specified	Not specified	[17]
Malvaceae	*Chiranthodendron pentadactylon* Lam.	Flor de manita, manita, mano de león. *In the state of Oaxaca: ita ndaa, tundakua*	Eye inflammation, eye problems	Flowers	Infusion	Toluca	[17]
Malvaceae	*Hibiscus rosa-sinensis* L.	Tulipán, obelisco, rehilete, tulipán fino tulipán rehilete, tulipán rojo	Eye infection	Not specified	Not specified	Not specified	[17]
Malvaceae	*Kearnemalvastrum subtriflorum* (Lag.) D.M.Bates	Malva macho	Cataracts	Root and leaves	Infusion	San Juan Tepecoculco, Estado de México	[14]
Malvaceae	*Malva* cf. *neglecta* Wallr.	Malva hembra	Cataracts	Whole plant	Infusion, local	San Juan Tepecoculco, Estado de México	[14]
Malvaceae	*Malva parviflora* L.	Malva, ahala, malba, malva de campo, malva de castilla, malva de quesitos. *In the state of Estado de México: du-ene (mazahua); in Oaxaca: baldag malv (zapoteco)*	Not specified	Not specified	Wash the eyes	Not specified	[17]
Malvaceae	*Triumfetta semitriloba* Jacq.	Cadillo, cadillo de abrojo, hierba de la soltura. *In the state of Nayarit: jaram (tepehuano); in Quintana Roo: moloch; in San Luis Potosí: thipaxi’ (tenek)*	Eye problems	Not specified	Not specified	Not specified	[17]
Marantaceae	*Maranta arundinacea* L.	Sagu, guajay platanillo, papatlitla, sagú de monte. *In the state of Quintana Roo: chaak; in San Luis Potosí: tsakam tdulup (tenek)*	Corneal opacity, blurred vision	Not specified	Not specified	Not specified	[17]
Melastomataceae	*Chaetogastra ferrariana* (Cogn.) P.J.F.Guim. & Michelang	*Scuan*	Eye irritation, eye pain	Flowers and fresh leaves	Powered flowers and fresh leaves applied locally	El Capulinar, Quimixtlán Puebla	[14]
Melastomataceae	*Chaetogastra ferrariana* (Cogn.) P.J.F.Guim. & Michelang	*Xani pogti (otomí)*	Eye irritation, rheumy eyes	Sap	Drops	San Pablito, Pahuatán, Puebla	[14]
Melastomataceae	*Chaetogastra tortuosa* (Bonpl.) DC.	*In the state of Puebla: konipotei (otomí)*	Eye infection, eye irritation	Not specified	Not specified	Puebla	[17]
Melastomataceae	*Miconia xalapensis* (Bonpl.) M. Gómez	Capulincillo, capulín, capulín agarroso, capulín de totola, hierba del coyote, palo de capulín de zorro. *In the state of Oaxaca: tzon te tzkoxiu. In Puebla: mchjut, nigua caolín, tesguate*	Eye inflammation, eye problems	Not specified	Not specified	Not specified	[17]
Moraceae	*Castilla elastica* Sessé	Hule, árbol de hule, caucho. *In the state of Oaxaca: oom; in Puebla: hullicuahuit, tzakat hulli; in Veracruz: tsacat; in Yucatán: k’iche’, k’ik’aban*	Eye irritation, corneal opacity, cataracts	Latex or sap	Not specified	Chiapas	[17]
Muntingiaceae	*Muntingia calabura* L.	Puan, capolín, capulín, puam, puanito. *In the state of Oaxaca: mujty; in Puebla: puguan (totonaco); in San Luis Potosí: puam, tsakam puwamte’ (tenek)*	Improving eyesight	Not specified	Not specified	Not specified	[17]
Myrtaceae	*Psidium guajava* L.	Guayaba, guava, guayabilla, guayabilla cimarrona, guayabo, guayabo morado, hojas de guayaba, palo de guayabo blanco, arena agria. *Xalxócotl*, *xaxokotl*, *xaxucotl (náhuatl)*	Eyes problems	Not specified	Not specified	Not specified	[17]
Onagraceae	*Oenothera pubescens* Wild. ex Spreng.	Linda tarde	Eye irritation	Not specified	Infusion, applied locally	San Rafael Tepatlaxco, Tlaxcala	[14]
Papaveraceae	*Argemone mexicana* L.	Chicalote espinoso	Eye irritation	Latex	Wash the eyes	Ahuatla, Quimixtlán, Puebla	[14]
Papaveraceae	*Argemone mexicana* L.	Chicalote	Eye pain, eye inflammation, stinging, corneal opacity, cataracts	Fresh latex, bark	Poultices prepared with the bark. Fresh latex is applied at night to the eyelid or inside the eye to relieve stinging, and mixed with mesquite juice (sp, n/r.), it is put into drops to remove corneal opacity from the eyes	Not specified	[17]
Papaveraceae	*Argemone mexicana* L.	Amapola espinosa, cardosanto, chicalote. *Chichicallotl*, *chicallot*	Conjunctivitis, rheumy eyes, cataracts, corneal opacity, fleshy growth of abnormal tissue of the conjunctiva	Latex	Latex mixed with milk is applied as eye drops daily before bedtime for conjunctivitis and rheumy eyes. To treat cataracts, one drop of the latex is applied directly to the eyes once in the morning. For corneal opacity and fleshy growths, the latex is mixed with water and applied directly to the eyes in three drops every day for one week. After this period, the drops are applied only every three days	Ciudad de México, Puebla and Oaxaca	[18]
Papaveraceae	*Argemone ochroleuca* Sweet	Cardo	Eye infection	Latex	Local	La Gloria, Anáhuac, Nuevo León	[14]
Papaveraceae	*Argemone ochroleuca* Sweet	*Cone (otomí)*	Eye irritation, eye infection	Latex	Local	San Pablito, Pahuatán, Puebla	[14]
Papaveraceae	*Argemone ochroleuca* Sweet	Chicalote	Eye pain	Latex	Local	Alpoyeca, Guerrero	[14]
Papaveraceae	*Argemone ochroleuca* Sweet	Chicalote	Cataracts	Root	Oral infusion	Huitel, Hidalgo	[14]
Papaveraceae	*Argemone ochroleuca* Sweet	Chicalote	Eye irritation	Latex	Local	San Miguel Regla, Hidalgo	[14]
Papaveraceae	*Argemone ochroleuca* Sweet	Amapola amarilla, cardo, chacalote, chicalote amarillo, espinocilla. *In the state of Michoacán: xate (purhépecha), shate, xata’; in Morelos: chiacalote, tlamexcaltzin; in Puebla: ko’ne potei (otomí)*	Eye irritation, eye infection, rheumy eyes	Flower, fruit	Apply the drops of juice that come from the flowers and fruits to the eyes	Puebla, Morelos, Michoacán, Durango, Oaxaca.	[17]
Papaveraceae	*Argemone ochroleuca* Sweet	Chicalote, amapola espinosa, cardo, cardosanto, flor espinosa. *Chichicalotl (nahua)*, *cone (otomí). In the state of Puebla: kone toeni (otomí); in Oaxaca: Yarg yech (zapoteco)*	Conjunctivitis, rheumy eyes, cataracts	Latex, stem	The latex or juice from the stems is applied as drops to the eyes once a day to treat conjunctivitis or rheumy eyes. For the treatment of cataracts, the juice of stems is placed on the affected eye every night	Ciudad de México, Durango, Estado de México, Guanajuato, Guerrero, Hidalgo, Jalisco, Michoacán, Morelos, Nuevo León, Oaxaca, Puebla, Querétaro, Sinaloa, Sonora, Tlaxcala, Zacatecas	[16]
Papaveraceae	*Argemone platyceras* Link & Otto	Chicalote	Eye problems	Latex	Local	Velasco, Hidalgo	[14]
Papaveraceae	*Argemone platyceras* Link & Otto	Chicalote	Expulsion of foreign body of the eye	Seeds	Apply the seed maceration with water to remove foreign body from the eyes	Michoacán	[17]
Papaveraceae	*Argemone sanguinea* Greene	Cardo	Cataracts	Latex	Local	Mina, Nuevo León	[14]
Papaveraceae	*Bocconia frutescens* L.	Gordolobo. *In the state of San Luis Potosí: ts’ixte’ (tenek)*	Eye pain	Not specified	Local	Not specified	[17]
Piperaceae	*Piper auritum* Kunth	Acoyo, acuyo, cordoncillo, cordoncillo blanco, hierba anís, hoja de cáncer, hoja santa, momo, yerba santa. *In the state of Oaxaca: ’ma jóo, ma ’haw, tzon tzko ntko, wo, woo; in Puebla: jinan (totonaco), coyoquelite, omequelite, omequilit-dos quelite, tlanepa, xalcuahuitl*	Eye problems	Not specified	Not specified	Not specified	[17]
Piperaceae	*Piper berlandieri* C. DC.	Cordoncillo. *In the state of Morelos: lalancualle (náhuatl); Oaxaca: pilin cos (huave), izcapatle*	Eye problems	Not specified	Not specified	Not specified	[17]
Pinaceae	*Pinus pseudostrobus* Lindl.	Ocote	Eye infection	Leaves	Grind to extract their juice. Drops	Michoacán	[17]
Plantaginaceae	*Mecardonia procumbens* (Mill.) Small	Escabiosa, flución; chotecte, quina; quita manchas de la cara. *In the state of San Luis Potosi: huitzil a k’iicha, tsakam wichab (tenek)*	Eye problems	Whole plant	Decoction	Guanajuato	[17]
Plantaginaceae	*Plantago major* L.	Llantén, anten, antena, cancerina, chile de pato, dianten, diasten, hierba del manzo, hojas de lantes, lanté, lantén, lanter, lantey, lengua de vaca, lenteja, lentem, liante, liantel, mucilago, planten, sábila, valeriana, yantén. *In the state of Chiapas: yok tje; in Michoacán: yurés xukuri (purhépecha); in Puebla: snoktail (totonaco); in Yucatán: xiw kin*	Eye inflammation, corneal opacity, rheumy eyes, fleshy growth of abnormal tissue of the conjunctiva	Not specified	Not specified	Michoacán, Morelos, Chiapas, Veracruz	[17]
Plantaginaceae	*Plantago major* L.	Llantén, lanté	Conjunctivitis, eye inflammation, tired eyes	Leaves	The leaves are boiled for 10 min and mixed with milk. Three drops are applied to the eyes daily	Ciudad de México	[18]
Plumbaginaceae	*Plumbago pulchella* Boiss.	Chililo, hierba del negro, pañete, pañiete, plumbago. *In the state of Morelos: itzcuinpantli*	Eye washer	Not specified	Not specified	Not specified	[17]
Plumbaginaceae	*Plumbago zeylanica* L.	Hierba de alacrán	Rheumy eyes	Leaves	Local. Leaves crushed	Culiacán Sinaloa	[14]
Poaceae	*Arundo donax* L.	Cañaveral, carrizo de sol. *In the state of Oaxaca: majchow, yag rstili; in San Luis Potosí: pakaab (tenek)*	Eye irritation, fleshy growth of abnormal tissue of the conjunctiva, corneal opacity	Buds	Wash eyes directly with the water from the buds	Puebla, Sonora, Durango	[17]
Portulacaceae	*Portulaca oleracea* L.	Verdolaga, faginera, mañita, seda, verdolaga de agua. *In the state of Michocán: tiangui; in Nayarit: venturagasha (cora); in Puebla: xpulh (totonaco), mixquilic, x’pul cac*	Eye inflammation	Not specified	Not specified	Not specified	[17]
Primulaceae	*Bonellia macrocarpa* subsp. *pungens* (A.Gray) B.Ståhl & Källersjö	San Juanico. *In the state of Sinaloa: t’aziiro (mayo)*	Eye problems	Leaves and fruits	Not specified	Sonora	[17]
Rosaceae	*Prunus serotina* var. *salicifolia* (Kunth) Koehne	Capulín, capulí. *In the state of Ciudad de México: det-sé (otomí), elocapolin (náhuatl);in Estado de México: deze (otomí); in Michoacán: shengua, xenhua (purhépecha); inSonora: jumpal (pima)*	Corneal opacity	Bark and leaves	Not specified	Not specified	[17]
Rosaceae	*Prunus serotina* var. *salicifolia* (Kunth) Koehne	Capulín. *Capolin*, *xegua*	Conjunctivitis, corneal opacity	Bark	The dried bark is crushed into a powder, which is then sprinkled over the eyes by blowing on it	Ciudad de México	[18]
Rosaceae	*Rosa centifolia* L.	Rosa de Castilla, rosa, rosa aromática, rosa blanca, rosa común, rosa de cien hojas, rosa té	Eye irritation, eye problems, wash the eyes, stinging	Flowers	Boiled	Not specified	[17]
Rosaceae	*Rosa centifolia* L.	Rosa de Castilla	Conjunctivitis, corneal opacity, tired eyes	Flowers, petals	The petals of two roses are placed in half a liter of boiling water. After 30 min, the liquid is filtered and used to wash the eyes daily	Ciudad de México	[18]
Rosaceae	*Rosa chinensis* Jacq.	Rosa de Castilla, rosa, rosa de guía. *In the state of Chiapas: roshohs chu’ulelal nichim roshosh*	Eye problems	Petals	Soak the petals in water overnight	Not specified	[17]
Rosaceae	*Rosa gallica* L.	Rosa de castilla, rosa blanca, rosa luto, rosa purga	Eye infection	Not specified	Not specified	Not specified	[17]
Rosaceae	*Rosa gallica* L.	Rosa blanca, rosa purga	Eye irritation	Flower	Local. Wash the eyes	Quimixtlán, Puebla	[14]
Rosaceae	*Rosa gallica* L.	Rosa de Castilla	Eye infection	Flower	Decoction. Local drops	Misantla, Veracruz	[14]
Rosaceae	*Rosa gallica* L.	Rosa de Castilla	Eye infection, eye irritation	Petals	For eye infections, drops of a decoction or infusion of the petals are applied to the eyes. Irritated eyes are washed with the infusion of the petals	Ciudad de México, Estado de México, Oaxaca, Puebla Veracruz	[16]
Rosaceae	*Rosa* sp.	Rosa de Castilla	Corneal opacity	Not specified	Not specified	Santa Catarina del Monte, Estado de México	[14]
Rutaceae	*Citrus aurantifolia* (Christm.) Swingle	Limón, lima, lima chichona, lima de chichi, lima de ombligo, limón agrio, limón liso, limonero. *In the state of Michoacán: cal-mu-nish, tsaj-pox; in Oaxaca: lium (huave); in Puebla: limunux (totonaco), limaj;; in San Luis Potosí: jiliy lemon (tenek)*	Eye problems, rheumy eyes	Fruit	Squeeze. Local	Not specified	[14]
Rutaceae	*Citrus limon* (L.) Osbeck	Limón, lima, limón chico, limón de tierra, limón grande, limón redondo, palo de limón dulce. *In the state of Guerrero: limoon; in Oaxaca*	Conjunctivitis	Not specified	Not specified	Not specified	[14]
Rutaceae	*Citrus limon* (L.) Osbeck	Limón	Conjunctivitis, styes, corneal opacity, glaucoma, trachoma	Fruit, juice	Squeeze the juice of 2 lemons into 1 glass of water and drink every morning on an empty stomach for 2 weeks. Rest for 1 week and continue until healed	Ciudad de México	[18]
Rutaceae	*Ruta chalepensis* L.	Ruda, lula. *In the state of Michoacán: aguitzearicua, akuiesi uarirakua, aku’ttsi usrhirakua, akuitze-huariracua (purhépecha)*	Eye problems	Not specified	Not specified	Not specified	[17]
Rutaceae	*Ruta chalepensis* L.	Ruda	Eye problems	Fresh branches	Local. Waving the herbs around the eyes	Juan Aldama, Zacatecas	[14]
Rutaceae	*Ruta chalepensis* L.	Ruda. *In the state of Michoacán:* ahuitse (purhé); *in Estado de México: kuan rud yixché (zapoteco); in Chiapas: lulá; in Puebla: roda (nahua)*	Conjunctivitis, excessive tearing	Leaves, whole plant	For excessive tearing, wash the eyes daily with a decoction or infusion of the leaves, twice a day. In the case of conjunctivitis, steam inhalations are made on the head and chest with a rue decoction before going to sleep, while keeping warm	Campeche, Chiapas, Ciudad de México, Estado de México, Guanajuato, Guerrero, Hidalgo, Michoacán, Morelos, Nayarit, Nuevo León, Oaxaca, Puebla, Querétaro, Sinaloa, Sonora, Tabasco, Tamaulipas, Tlaxcala, Veracruz, Zacatecas	[16]
Rutaceae	*Ruta chalepensis* L.	Ruda	Conjunctivitis, excessive tearing	Leaves	Boil 100 g of leaves in half a liter of water for 25 min. Wash the eyes daily until healed	Ciudad de México	[18]
Sapindaceae	*Cardiospermum halicacabum* L.	Alfombrilla, bejuco, farolitos, hierba del golpe, tronador. *In the state of San Luis Potosí: ejtil tsankub ts’ojol (tenek)*	Eyes problems	Not specified	Not specified	Not specified	[17]
Sapindaceae	*Paullinia clavigera* Schltdl.	*Tlancoismecatl (náhuatl)*	Not specified	Sap	Eye washer. Drops	Tepenehuac, Veracruz	[14]
Sapindaceae	*Serjania racemosa* Schum	Bejuco tres un uno, hierba del golpe, bejuco siete corazones, nueve hojas, vara de tres costillas. *In the state of Guerrero: yucu golpe (mixteco); in Puebla: takakatsidik (tepehua)*	Eye problems	Leaves	Add salt, put over the fire, after a short time add aguardiente (an alcoholic beverage). Apply to the affected area	Not specified	[17]
Scrophulariaceae	*Buddleja americana* L.	Hierba del mosco, hierba del pasmo, tabaco silvestre, teposan, tepuz, tepuza, topozán, zompantle. *In the state of Chiapas: sitít, tze-lepat, tzelepetl; in Hidalgo: shopochíhuitl; in Michoacán: mitziwa; in Veracruz: cayolican, coyolican; in San Luis Potosí: tdak te’, pulik elte’ (tenek)*	Eye inflammation	Not specified	Not specified	Not specified	[17]
Solanaceae	*Capsicum annuum* L.	Chile; chiltepín, vhile amachito, chile chiltepín, chile chipotle, chile verde, mata de chilar chiltepé. *In the state of Oaxaca: tzon tz a kitza; in Puebla: acxispin (tepehua), akgtsispin, pin (totonaco); in Veracruz: stilampin; in Yucatán: chakik, ya’ax ik; in San Luís Potosí: its (tenek)*	Eye pain	Not specified	Not specified	Not specified	[17]
Solanaceae	*Jaltomata procumbens* (Cav.) J.L.Gentry	Jitomate negro. *In the state of Estado de México: jaltomat; in Tlaxcala: coscomate, coscotomate*	Eye problems	Not specified	Not specified	Not specified	[17]
Solanaceae	*Nicotiana tabacum* L.	Tobacco. Tabaco, tabaco cimarrón, tabaco de la montaña. *In the state of Chiapas: anjel, ch’ul winik, moy (tzotzil), may, may wamal (tzeltal); in Michoacán: tabaku; in Morelos: picietl, tenexiet (náhuatl), piciete, quahyetl, tenejiete;*	Eye pain, eye problems	Not specified	Not specified	Not specified	[17]
Solanaceae	*Nicotiana tabacum* L.	Tobacco. Tabaco	Eye problems	Leaves. Smoke	Local	Misantla, Veracruz	[14]
Solanaceae	*Physalis coztomatl* Moa & Sessé	Costomate, tomate agrio, tomate amarillo, tomatillo, tomate, tomatito de hoja. *In the state of Morelos: coztomatl*	Corneal opacity	Not specified	Not specified	Not specified	[17]
Solanaceae	*Physalis philadelphica* Lam.	*In the state of Ciudad de México: miltómatl (náhuatl)*	Eye problems	Not specified	Not specified	Not specified	[17]
Solanaceae	*Physalis pubescens* L.	Cáscara de tomate, tomate, tomate de hoja. *In the state of Estado de México: costomate*	Corneal opacity	Not specified	Not specified	Not specified	[17]
Solanaceae	*Solanum americanum* Mill.	Hierba mora, hierba mora blanca, hoja de zalazar, quelite mora, yerbamora. *In the state of Chiapas: moen (tzeltal), muil itaj, mu mu itai, unem mu, yerma mora (tzotzil); in Morelos: totunchichic; in Oaxaca: yiwa tii (mixteco)*	Corneal opacity	Not specified	Not specified	Not specified	[17]
Solanaceae	*Solanum lycopersicum* L.	Tomato. Jitomate, coatomate, cuatomate, toma, tomate. *In the state Puebla: pach (tepehua), palucha; in Quintana Roo: ahp’ak, beyanchan, pak, tsululep’ak, tsunuup’ak (maya); in Veracruz: paklhshu, siwapaklhsu; in San Luis Potosí: tutdey, tudhay (tenek)*	Corneal opacity	Juice	Not specified	Not specified	[17]
Solanaceae	*Solanum nigrescens* Mart. & Galeotti	Hierba mora, cielo raso, hierbamora, mora, rama de la mulata, vara de cuete, yerba mora. *In the state of Michoacán: tsikutakua (purhépecha), kuatsitasi; in Morelos: tohtomatic (náhuatl); in Puebla: ix pakgcha tontsiktsi (totonaco), mustulk (tepehua), pipisconchi, té conchichi*	Eye pain	Juice of the whole plant	Apply drops into the eyes	Not specified	[17]
Solanaceae	*Solanum nigrum* L.	Hierba mora. *Tomaquilitl (náhuatl)*	Eye problems	Branches	Wash the eyes with the water from the boiled branches	Xolotla, Pahuatlán, Puebla	[14]
Solanaceae	*Solanum nigrum* L.	Hierba mora, chichiquelite, hoja de salazar, jaltomate, mambia, quebra plato quelite, tomatillo, tumiar, veneno de cuervo, yerba mora. *In the state of Estado de México: denshá, den-shojo (mazahua), ra roska (otomí), toonchichi; in Guerrero: yicua tii (mixteco); in Michoacán: potsekuaricho (purhépecha)*	Corneal opacity, eye pain	Not specified	Not specified	Not specified	[17]
Solanaceae	*Solanum rostratum* Dunal	Duraznillo, abrojo, colmillo de puerco, espina amarilla, espinaca del cerro, flor de duraznillo, hierba de la manca mula, hierba del sapo, huevo de chucho, mala mujer, manca mula, mata yegua. *In the state of Morelos: ayhuixcle; in Oaxaca: guiici baloo (zapoteco); in Puebla: iztecuate*	Fleshy growth of abnormal tissue of the conjunctiva, corneal opacity	Branches	Decoction	Not specified	[17]
Solanaceae	*Solanum tuberosum* L.	Papa	Fleshy growth of abnormal tissue of the conjunctiva, corneal opacity	Tuber	Slice and leave on the eyelids overnight	Michoacán	[17]
Solanaceae	*Solanum tuberosum* L.	Papa	Eye inflammation, eyelid inflammation, tired eyes, eye pain, inflammation of tear ducts	Tuber	Slice and leave on the eyelids overnight	Ciudad de México	[18]
Tropaeolaceae	*Tropaeolum majus* L.	Mastuerzo	Eye problems	Flowers	Wash the eyes with the flower infusion	San Juan Tepecoculco, Estado de México	[14]
Verbenaceae	*Aloysia citrodora* Paláu	Cedrón	Eye pain, eye problems	Not specified	Not specified	Not specified	[17]
Verbenaceae	*Lippia dulcis* Trevir.	Hierba dulce	Eye infection	Not specified	Not specified	Tamazunchale, San Luis Potosí	[14]
Viburnaceae	*Sambucus mexicana* C.Presl ex DC.	Saúco	Eye irritation	Leaves	External, to clean the eye	Quimixtlán, Puebla	[14]
Viburnaceae	*Sambucus mexicana* C.Presl ex DC.	Sauco, alcanfor, flor de sauco, guarico, sauce, sauco chico, sauco grande, sauzo, tapiro. *In Nayarit: joday llochic (tepehuano); in Puebla: tongatsi (otomí), toqiwa, topsiwi (totonaco), xumetl (náhuatl), tengatsi, xomet; in San Luis Potosí: sawko (tenek); in Veracruz: azumate, azumatl, toxiwua; toxio (tepehua), s’auco (zoque-popoluca)*	Conjunctivitis, eye infection	Not specified	Not specified	Not specified	[17]
Vitaceae	*Ampelocissus erdvendbergianus* Planch.	*Komyuuxy (mixe)*	Eye infection	Latex	Local	San Juan Guichicovi, Oaxaca	[14]
Vitaceae	*Cissus verticillata* subsp. *verticillata*	Sanalotodo, tripa de Judas, alquilón, bejuco, bejuco de sanalotodo, bejuco loco, cola del diablo, hierba del buey, jiote, rondón, sana todo, secapalo, uva silvestre, tripa del diablo, tripa de vaca, tripa de Judas. *Temecatlxihuitl (náhuatl)*	Corneal opacity		Decoction		[17]
Vitaceae	*Vitis tiliifolia* Humb. & Bonpl. ex Schult.	Barra Negra	Eye infection	Sap	Local. Drops	Misantla, Veracruz	[14]
Vitaceae	*Vitis tiliifolia* Humb. & Bonpl. ex Schult.	Parra, bejuco blanco, parra de bejuco, parra negra, siete corazones, tripa del diablo, uva, uva de campo, uva de monte. *In the state of Puebla: cuaxtamepu (otomí), parrasmescat (náhuatl), snu ’jut (totonaco); in Veracruz: yec-tm-say*	Eye infection, eye irritation, eye problems	Not specified	Not specified	Veracruz, Puebla	[17]
Zingiberaceae	*Zingiber officinale* Roscoe	Ginger. Ajenjibre, jengibre. engible, gengibre, injible. *In the state of Puebla: castilanchile (náhuatl), caxta lam’pin, tonahuiz camu (totonaco), ixthi, nixtí (otomí)*	Blindness	Not specified	Not specified	Not specified	[17]
Zygophyllaceae	*Guaiacum sanctum* L.	Guayacán, guácima, guázuma. *In the state of Quintana Roo: chun chintok, soon, soon chulul (maya)*	Improving eyesight	Not specified	Not specified	Not specified	[17]

## Data Availability

No new data were created or analyzed in this study. Data sharing is not applicable to this article.
